# ZmMYB127 Modulates Maize Kernel Texture and Size by Integrating the Synthesis of Starch, Zein Proteins and Auxin

**DOI:** 10.1111/pbi.70384

**Published:** 2025-09-24

**Authors:** Tiandan Long, Yayun Wang, Zhou Liu, Yongbin Wang, Changqing Mao, Dening Wang, Aying Qin, Qiang Liao, Jin Yang, Xiujun Fan, Lei Gao, Yufeng Hu, Jing Wang, Yubi Huang, Yangping Li

**Affiliations:** ^1^ State Key Laboratory of Crop Gene Exploration and Utilization in Southwest China Sichuan Agricultural University Chengdu Sichuan China; ^2^ Crop Research Institute Hunan Academy of Agricultural Sciences Changsha Hunan China; ^3^ College of Agronomy Sichuan Agricultural University Chengdu Sichuan China; ^4^ Industrial Crop Research Institute Sichuan Academy of Agricultural Sciences Chengdu Sichuan China

**Keywords:** auxin, maize, starch, vitreous endosperm, zein proteins, ZmMYB127

## Abstract

Kernel texture is an important agronomic trait that determines the end‐uses of maize kernels and affects their integrity at harvest and susceptibility to pests and diseases. The ratio of the vitreous endosperm (RVE) is the key index for assessing kernel texture, and identifying key genes involved in its formation is crucial for maize breeding. Here, through genome‐wide association study (GWAS), haplotype analysis and transgenic kernels phenotyping, we characterised ZmMYB127, an endosperm‐specific R2R3‐MYB transcription factor, which positively regulates vitreous endosperm (VE) formation. *ZmMYB127* is preferentially expressed in VE cells during the filling stage and antagonistically regulates zein protein and starch synthesis in the endosperm. Notably, ZmMYB127 interacts with OPAQUE2 (O2) to synchronously transactivate genes encoding α‐zein proteins and interacts with prolamin‐box binding factor 1 (PBF1) to additively suppress genes involved in starch synthesis, thereby governing kernel texture. Moreover, ZmMYB127 negatively regulates genes involved in indole‐3‐acetic acid (IAA) synthesis in the endosperm, affecting endosperm development and size, thereby linking the function of ZmMYB127 to kernel size. In conclusion, our study unravels the transcription factor ZmMYB127 that modulates kernel texture and size by integrating regulation of starch, zein and auxin synthesis pathways in maize endosperm. Additionally, our findings provide valuable genetic resources for breeding or engineering maize varieties with improved kernel texture and quality.

## 1 Introduction

Maize ( *Zea mays* L.) is a globally significant staple food and feed crop, with kernel texture being a key agronomic trait that affects its applications in food, feed and industry. Endosperm vitreousness, a crucial quality attribute of kernel texture, is typically determined by the ratio of vitreous endosperm (RVE) in the peripheral region to starchy endosperm (SE) in the centre (Fu et al. 2023; Gibbon and Larkins 2005). Kernels with higher RVE are denser and have higher test weight, contributing to the resistance to physical damage during harvesting, storage and transportation (Wang et al. 2020; Zhang and Xu 2019). Moreover, high‐RVE kernels are less susceptible to pests and fungal infections, improving both industrial efficiency and processed food quality. In contrast, SE is characterised by opaque, floury and fragile, making it more vulnerable to pests and diseases (Fu et al. 2023; Wang et al. 2020; Zhang et al. 2011).

The formation of vitreous endosperm (VE) has been extensively studied, with the compactness of the starch‐protein matrix recognised as a critical determinant (Gayral et al. 2016). Starch and storage proteins, mainly zeins, are the dominant components in the endosperm, largely determining both yield and quality of maize. During endosperm filling, starch accumulates as starch granules (SGs) within amyloplasts, while zeins are deposited in protein bodies (PBs) (Hannah and Boehlein 2017; Larkins et al. 2017). In the cytoplasm, SGs are surrounded by PBs and amorphous proteins. The peripheral endosperm cells are predominantly filled with PBs and SGs, while the central endosperm cells primarily contain SGs with few PBs. During the maturation phase, the condensation of these components leads to the formation of VE and SE (Fu et al. 2023; Gibbon and Larkins 2005; Wang et al. 2020). Therefore, VE is characterised by close‐packed SGs with a compact protein matrix and SE features spherical SGs with a discontinuous protein matrix (Wang et al. 2020; Xu et al. 2019; Zhang and Xu 2019).

The synthesis of starch and zeins in maize has been well characterised. Starch synthesis in the endosperm requires the synergistic activity of multiple key enzymes, including sucrose synthase (SUS), adenosine diphosphoglucose pyrophosphorylase (AGPase), ADP‐glucose transporter (BT1), starch synthase (SS), starch branching enzyme (SBE), starch debranching enzyme (DBE) and starch phosphorylase (SP) (Hannah and Boehlein 2017; Huang, Tan, et al. 2021). Extensive research has shown that core starch synthesis genes (SSGs), which are specifically or highly expressed in the endosperm during the filling stage, determine the final starch content and structure (Hu et al. 2021; Huang, Tan, et al. 2021; Zhang et al. 2016). Zeins are categorised into α‐ (19‐kD z1A, z1B and z1D; 22‐kD z1C), β‐ (15‐kD), γ‐ (50‐kD, 27‐kD and 16‐kD), and δ‐zeins (18‐kD and 10‐kD) based on their structure and molecular mass (Yang et al. 2023). The maize genome contains multiple copies of genes encoding α‐zeins, while other zein genes occur as single copies. Zeins are the primary storage proteins in the endosperm, accounting for nearly 50% of the total transcripts (Chen et al. 2014; Yang et al. 2023). Numerous mutants affecting zein and starch synthesis have been found to affect VE formation in maize (Larkins et al. 2017; Wang et al. 2020).

Starch and protein accumulation in maize endosperm is primarily regulated at the transcriptional level, involving a series of *cis*‐acting motifs and transcription factors (TFs) (Huang, Tan, et al. 2021; Li et al. 2021; Yang et al. 2023). Maize endosperm‐specific bZIP transcription factor OPAQUE2 (O2) recognises the O2‐box to transactivate nearly all zein genes, as well as pyruvate orthophosphate dikinases (PPDKs) and starch synthase III (SSIII), which are critical components of the starch biosynthetic enzyme complex, highlighting its central role in endosperm filling and development (Li et al. 2015; Schmidt et al. 1987; Zhan et al. 2018; Zhang et al. 2015, 2016). Prolamin‐Box Binding Factor (PBF1), another well‐known DOF family TF, physically interacts with O2 to co‐regulate the expression of SSGs and zein genes by recognising the prolamine box (P‐box) in their promoters (Vicente‐Carbajosa et al. 1997; Zhang et al. 2015, 2016). Mutations in *O2* and *PBF1* result in drastically reduced zein levels, leading to an opaque and SE phenotype (Schmidt et al. 1987; Vicente‐Carbajosa et al. 1997; Zhang et al. 2016), indicating that transcriptional regulators which control starch and zein protein synthesis would regulate the formation of VE. Although studies on opaque mutants provided valuable insight into the crucial role of starch and zein proteins in the formation of VE, the key genes responsible for endosperm vitreousness and the regulatory mechanisms remain to be further investigated.

Phytohormones play important roles in endosperm development and filling. Among them, auxin is the only endogenous hormone that maintains high levels throughout the filling stage in maize endosperm (Doll et al. 2017; Locascio et al. 2014; Lur and Setter 1993). Indole‐3‐acetic acid (IAA), the primary natural auxin, is predominantly synthesised via the tryptophan‐dependent indole‐3‐pyruvic acid (IPA) pathway (Chourey et al. 2010; Song et al. 2024). *Defective18* (*De18*, also known as *ZmYUC1*) is responsible for the endosperm‐specific IAA synthesis in maize, and the mutant *de18* exhibits reduced endosperm cell division and kernel size (Bernardi et al. 2012, 2019; Locascio et al. 2014). Moreover, reduced IAA accumulation in the pericarp affects maize kernel architecture, resulting in shorter and denser kernels (Wang et al. 2024). Recent spatial transcriptomics show that genes involved in IAA synthesis are highly expressed in both VE and SE (Fu et al. 2023). Moreover, ZmNAC128/130 has been found to coordinate the synthesis of major storage reserves and IAA in maize endosperm (Song et al. 2024), indicating that some genes within the regulatory network are regulated by common TFs. Despite the crucial role of auxin in endosperm development, its regulatory mechanisms in maize remain poorly understood.

To identify potential key genes involved in endosperm vitreousness, we performed a genome‐wide association study (GWAS) involving 238 inbred maize lines, identifying ZmMYB127, an endosperm‐specific R2R3‐MYB TF, as being associated with RVE. Phenotype analysis of transgenic lines confirmed that ZmMYB127 acts as a positive regulator of VE formation. Molecular analysis revealed that ZmMYB127 antagonistically regulates starch and zein synthesis to modulate VE formation. We demonstrated that ZmMYB127 interacts with PBF1 to additively suppress the expression of *Bt1* and *Ae1*, which are associated with starch synthesis, while also interacting with O2 to synergistically promote the expression of *z1D2* and *z1C1*, related to zein synthesis. Additionally, ZmMYB127 regulates the expression of *De18* and *TAR1*, two genes essential for IAA synthesis in maize endosperm, thereby modulating endosperm development and size. Taken together, our findings demonstrate that ZmMYB127 regulates kernel texture and size by integrating the synthesis of storage reserves and auxin in maize endosperm and providing valuable insights into the mechanisms of VE formation.

## 2 Results

### 2.1 Identification of ZmMYB127 Associated With Endosperm Vitreousness in Maize by GWAS

To evaluate the endosperm vitreousness of mature kernels, we collected samples from 238 inbred lines grown in three environments: Chongzhou in 2019 (2019CZ), Xinxiang in 2019 (2019XX) and Chongzhou in 2020 (2020CZ). We measured the relative VE area of mature kernels via multi‐threshold segmentation technology (Figure S1 and Table S1). Our analysis revealed significant variation in RVE among the inbred lines across all three environments, with values ranging from 16.1% to 79.45% (2019CZ), 10.12% to 70.00% (2019XX) and 14.77% to 74.53% (2020CZ) (Figure S2a and Table S2). Furthermore, comparisons among different subgroups indicated that the inbred lines from the Iodent (Iowa experiment station Reid's yellow dent) subgroup exhibited significantly lower RVE values than those from other subgroups in all three environments, while the SPT (Si ping tou) subgroup had the highest RVE values (Figure S2b).

**FIGURE 1:**
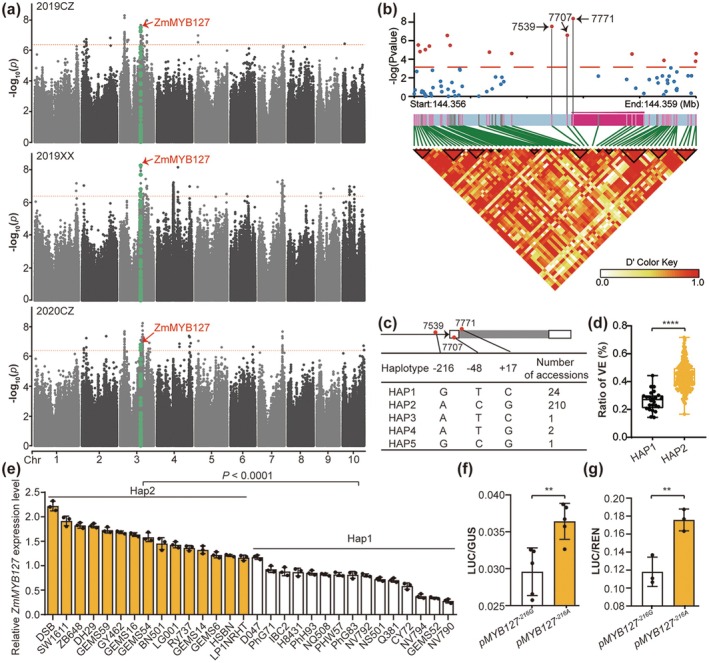
Genome‐wide association study (GWAS) identifies *ZmMYB127* as a candidate gene strongly associated with maize endosperm vitreousness. (a) Manhattan plots showing the associations of SNPs with the ratio of vitreous endosperm (RVE). The dashed horizontal line indicates the Bonferroni‐adjusted significance threshold (*p* < 4.11 × 10^−7^). The SNPs shown as green dots are located within the *ZmMYB127* gene. (b) Association analysis of sequence variants in the 2000‐bp upstream and downstream regions of *ZmMYB127* for the RVE in 238 inbred lines. The three most significant polymorphisms (7539, 7707 and 7771) were in strong linkage disequilibrium and were indicated by black arrows. The gene structure of *ZmMYB127* was shown below the association analysis plot. (c) Haplotype classification of three selected SNPs. (d) Boxplot showing the RVE of Hap1 (GTC, *n* = 24) and Hap2 (ACG, *n* = 210). The box shows the median and interquartile range, and the whiskers extend to the maximum and minimum values. (e) The expression analysis of *ZmMYB127* in 15 randomly selected lines from HAP1 and HAP2, respectively. Data are presented as the mean ± SD (*n* = 3). (f, g) Transient expression assay of promoter activity in maize endosperm at 10 days after pollination (DAP) via particle bombardment (f, *n* = 6) and in maize leaf protoplasts (g, *n* = 3). Statistical significance (**p < 0.001; ****p < 0.0001) was determined by a two‐tailed Student's *t* test, as shown in (d–g).

We conducted a GWAS using 2 432 795 high‐quality SNPs derived from 10× whole‐genome resequencing and RVE phenotypic data across the three environments. Using the FarmCPU model for association analysis, we identified a total of 309 significant SNPs (*p* < 4.11 × 10^−7^) associated with RVE across the whole association panel (Figure S2c and Figure 1a), of which 72, 201, and 80 significant SNPs with the highest −log_10_ (*p*) values of 8.29, 8.15 and 8.22 were identified in 2019CZ, 2019XX and 2020CZ, respectively (Figure 1a). Finally, we identified 14 significant loci, corresponding to 29 candidate genes that were tightly associated with endosperm vitreousness in at least two environments (Table S3). Notably, a significant locus on chromosome 3 (qRVE3‐2) was identified as strongly associated with RVE in all three environments, with all significant SNPs within this locus linked to the same candidate gene, *ZmMYB127* (Figure 1a and Table S3).

Furthermore, we performed an association analysis of SNPs within the coding sequence (CDS) and 2000 bp upstream/downstream regions of *ZmMYB127*, based on the RVE‐best linear unbiased estimate (BLUE) values, identifying 69 SNPs (Figure 1b). We further investigated the contributions of these SNPs to RVE variations through association analysis. Three adjacent SNPs (S3_144357539 in the promoter region, S3_144357707 in the 5′‐UTR, and S3_144357771 in the exon region) were found to be the most significant SNPs associated with RVE (Figure 1b,c), exhibiting strong linkage disequilibrium within the maize population (*r* ^2^ = 0.93). Based on the variation in these SNPs, we categorised 238 maize inbred lines into five haplotypes (HAP1‐5) (Figure 1c and Table S4). Among these, HAP1 (GTC) and HAP2 (ACG) were the two major haplotypes (*n* ≥ 5), containing 24 and 210 accessions, respectively. Phenotypically, members of HAP2 showed significantly greater RVE compared to those of HAP1 (Figure 1d), with no obvious difference in 100‐kernel weight between the two haplotypes (Figure S3a and Table S4). Moreover, transcriptome data from previous studies (Fu et al. 2013; Liu et al. 2017) indicated that *ZmMYB127* expression in HAP2 kernels at 15 DAP was significantly higher than in HAP1 kernels (Figure S3b and Table S4). We further randomly selected 15 inbred lines from each haplotype and analysed *ZmMYB127* expression by RT‐qPCR, which confirmed higher expression in HAP2 than in HAP1 (Figure 1e). Consistent with this result, transient expression assays revealed that the activity of the *ZmMYB127* ^*−216A*^ (HAP2) promoter was significantly greater than that of the *ZmMYB127* ^*−216G*^ (HAP1) promoter (Figure 1f,g). These results strongly indicate that natural variations in *ZmMYB127* are highly associated with maize endosperm vitreousness through alterations in gene expression.

### 2.2 Functional Validation of ZmMYB127

**FIGURE 2:**
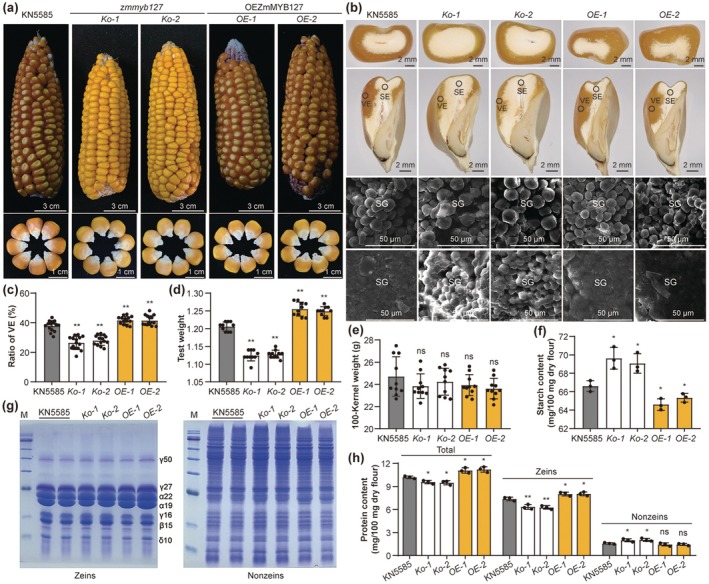
Genetic verification and biochemical analysis of ZmMYB127. (a) Ear and kernel phenotypes of the KN5585, *zmmyb127* knockout lines (*Ko‐1* and *Ko‐2*) and *ZmMYB127*‐overexpression lines (*OE‐1* and *OE‐2*) in the KN5585 background. (b) Transverse and longitudinal sections of mature wild type (WT) KN5585, knockout and overexpression line kernels, along with scanning electron micrographs of longitudinal sections showing the enlarged starchy endosperm area (SE, upper panel) and vitreous endosperm area (VE, lower panel), respectively. SG, starch granule. (c–e) Measurement of the RVE (c), test weight (d) and 100‐kernel weight (e) of the WT KN5585, knockout and overexpression lines. (f) Measurement and comparison of starch content in mature kernels of the WT KN5585, knockout and overexpression lines. (g) SDS‐PAGE analysis of zein (left) and nonzein (right) proteins in mature kernels of the WT KN5585, knockout and overexpression lines. The total protein loaded in each lane was equivalent to 400 μg of mature kernel flour. The size of each zein protein band is indicated beside it: γ50, 50‐kD γ‐zein; γ27, 27‐kD γ‐zein; α22, 22‐kD α‐zein; α19, 19‐kD α‐zein; γ16, 16‐kD γ‐zein; β15, 15‐kD β‐zein; δ10, 10‐kD. M, protein marker. (h) Measurement and comparison of protein content (including total, zein and nonzein proteins) in mature kernels of the WT KN5585, knockout and overexpression lines. Data are presented as the mean ± SD. Statistical significance (ns, not significant; *p < 0.05; **p < 0.01) was determined by a two‐tailed Student's *t* test, as shown in (c–f, h).

To investigate the biological function of ZmMYB127 in regulating endosperm vitreousness, we generated knockout mutants in the KN5585 inbred line using the CRISPR‐Cas9 system. The guide RNA target site was designed to edit the exon near the ATG. We identified two independent knockout lines, *Ko‐1* and *Ko‐2*, that harboured a 5‐bp deletion and a 1‐bp deletion, respectively. Both mutations led to early frame shifts and premature stop codons (Figure S4a). Heterozygous (*Ko‐1*/+ and *Ko‐2*/+) and homozygous plants (*Ko‐1* and *Ko‐2*) lacking the CRISPR/Cas9 construct were selected and self‐pollinated. Representative ears from each self‐pollinated line are shown in Figure 2a and Figure S4b. The ears of heterozygous plants segregated into opaque and normal kernels at a 1:3 ratio (*Ko‐1*/+, 56:170; *Ko‐2*/+, 66:200), while the homozygous plants produced only opaque kernels. To perform the allelic test, we crossed *Ko‐1* and *Ko‐2*, which resulted in ears showing no complementation of the mutant phenotype, confirming that *Ko‐1* and *Ko‐2* are allelic (Figure S4c). Notably, these alleles produced larger kernels compared to the wild type (WT) KN5585 (Figure 2a and Figure S4b–d). Statistical analysis revealed that the length and thickness of the *zmmyb127* kernels significantly increased, while their kernel width remained unchanged (Figure S4e–g).

We also developed transgenic lines that overexpress *ZmMYB127* driven by the *Ubiquitin* (*Ubi*) promoter in the KN5585 background. Two independent overexpression lines, *OE‐1* and *OE‐2*, exhibited significantly increased expression levels of *ZmMYB127* (Figure S5a) and demonstrated stronger light transmission in a light box, along with reduced kernel size (Figure 2a and Figure S5b,c). Compared to WT KN5585 kernels, the *ZmMYB127‐OE* kernels showed significantly decreased length and width, while the kernel thickness remained unchanged (Figure S5d–f). Examination of transverse and longitudinal sections revealed that the *Ko‐1* and *Ko‐2* kernels had fewer VE regions in the peripheral area compared to WT KN5585 kernels, whereas the two *ZmMYB127‐OE* lines exhibited larger VE regions (Figure 2b). Specifically, the *Ko‐1* and *Ko‐2* kernels had 31% and 25% less VE than WT kernels, while the *OE* lines had 11% and 10% more VE (Figure 2c). Consistent with the RVE values, the test weights of the *Ko‐1* and *Ko‐2* kernels significantly decreased, while those of *OE‐1* and *OE‐2* kernels increased (Figure 2d). However, no significant differences in 100‐kernel weight at maturity were observed among the WT, *Ko* and *OE* lines (Figure 2e).

Scanning electron microscopy (SEM) analysis of the SE region and the VE region of the kernels revealed that *Ko‐1* and *Ko‐2* lines had larger SGs in the SE region compared to WT and *OE* lines. SGs in the VE region of the *Ko‐1* and *Ko‐2* lines were embedded in a less proteinaceous matrix than in the WT and *OE* lines (Figure 2b). We determined the starch content in mature kernels based on dry whole‐grain flour, finding higher starch content in *Ko‐1* and *Ko‐2* lines (4.5% and 3.7% more than WT), while *OE‐1* and *OE‐2* lines had lower starch content (3.0% and 1.9% less, respectively) (Figure 2f). Additionally, we extracted zeins and nonzeins from mature kernels and conducted SDS–PAGE and biochemical analyses; the results revealed that *Ko‐1* and *Ko‐2* lines exhibited a substantial reduction in zein content compared to WT, particularly the 19‐kD α‐zeins (Figure 2g,h). Given that zein constitutes ~60% of the total protein content in the endosperm, the slight increase in nonzein protein levels did not compensate for the overall decrease. Consequently, the total protein content was lower in the *Ko* lines. In contrast, the zein and total protein contents in the *OE* lines significantly increased, while nonzein content did not show a significant difference between WT and *OE* lines (Figure 2h).

Additionally, we generated knockout mutants in the B104 inbred line and identified a CRISPR/Cas9‐generated mutant with a 1‐bp insertion, designated *Ko‐B1* (Figure S6a). This mutant displayed similar phenotypes to the *Ko* lines in the KN5585 background, characterised by opaque and large kernels, with *Ko‐B1* kernels appearing completely nonvitreous. The peripheral region of B104 endosperm is vitreous, whereas the *Ko‐B1* endosperm is entirely starchy (Figure S6b–h). Moreover, the *Ko‐B1* kernels exhibited lower levels for most amino acids, but total lysine content, which is deficient in zeins, was significantly increased in these kernels (Figure S7a). Most free amino acids in the *Ko‐B1* kernels were lower than in B104 kernels, while free proline content was three times higher in *Ko‐B1* compared to B104 (Figure S7b).

### 2.3 ZmMYB127 Is an Endosperm‐Specific R2R3‐MYB TF

**FIGURE 3:**
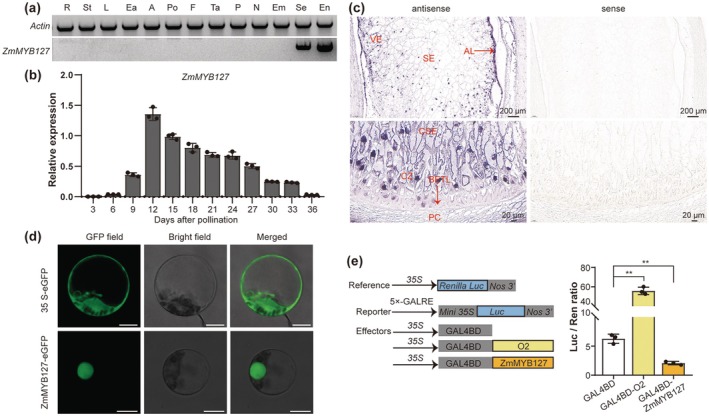
Expression and functional characteristics of ZmMYB127. (a) Semiquantitative RT–PCR analysis of *ZmMYB127* gene expression in different tissues of maize. A, anther; Ea, ear; Em, embryo; En, endosperm; F, filament; L, leaf; N, nucellus; P, pericarp; Po, pollen; R, root; Se, seed; St, stem; Ta, tassel. (b) RT–qPCR analysis of *ZmMYB127* expression in the developing kernel. Data are presented as the mean ± SD (*n* = 3). (c) RNA in situ hybridization of *ZmMYB127* in 12‐DAP kernels. Antisense probes were used to detect the spatial expression of *ZmMYB127* transcripts. Sense probes were used as negative controls. AL, aleurone; BETL, basal endosperm transfer layer; CSE, central starchy endosperm; CZ, conducting zone; PC, placento‐chalazal region; SE, starchy endosperm; VE, vitreous endosperm. (d) Subcellular localization of ZmMYB127 in maize leaf protoplasts. *35S‐eGFP* was used as the control. GFP, green fluorescent protein. Bar = 10 μm. (e) The transcriptional analysis of ZmMYB127 in maize leaf protoplasts. The left panel is a vector construction diagram. LUC, firefly luciferase; 35S, CaMV 35S promoter; Ren, Renilla Luc. The ratio of Luc/Ren in the right panel represents the activity of ZmMYB127. Data are presented as the mean ± SD (*n* = 3). Statistical significance (**p < 0.01) was determined by two‐tailed Student's *t*‐test.

Protein sequence analysis revealed that ZmMYB127 comprises 263 amino acids, featuring two SANT domains characteristic of the R2R3‐MYB subfamily, with no additional conserved domains identified (Figure S8a,b). We next constructed a phylogenetic tree using ZmMYB127 and its homologues from other species. The analysis revealed that ZmMYB127 is highly conserved among monocots and exhibits close phylogenetic relationships with TaMYB44‐4D in wheat and OsMYB44 in rice (Figure S8c). Semi‐quantitative RT‐PCR analysis in various maize tissues revealed that *ZmMYB127* is expressed specifically in the endosperm (Figure 3a). Subsequent RT‐qPCR analysis confirmed that its expression remains high from 9 to 27 days after pollination (DAP), peaking at 12 DAP (Figure 3b), aligning with an updated maize gene atlas (Figure S9a) (Hoopes et al. 2019). Additionally, RNA in situ hybridization showed abundant *ZmMYB127* expression in VE cells and notable signals in SE cells, the aleurone layer (AL) and the conducting zone (CZ), while only residual signals were observed in the basal endosperm transfer layer (BETL) (Figure 3c). This spatial distribution is consistent with public RNA‐Seq data (Figure S9b,c) (Fu et al. 2023; Zhan et al. 2015).

To investigate the functional properties of ZmMYB127, we assessed its subcellular localization and transactivation capacity. The full‐length *ZmMYB127* gene was fused with enhanced GFP (eGFP) and expressed under the constitutive *35S* promoter in both maize leaf protoplasts and onion epidermal cells. Unlike free eGFP, ZmMYB127‐GFP signals were exclusively localised in the nucleus (Figure 3d and Figure S10a). The yeast transactivation assay indicated that ZmMYB127 lacks transcriptional activity (Figure S10b), and the dual‐luciferase reporter (DLR) assay further confirmed that ZmMYB127 functions as a transcriptional repressor (Figure 3e).

### 2.4 ZmMYB127 Differentially Regulates Starch and Zein Synthesis

**FIGURE 4:**
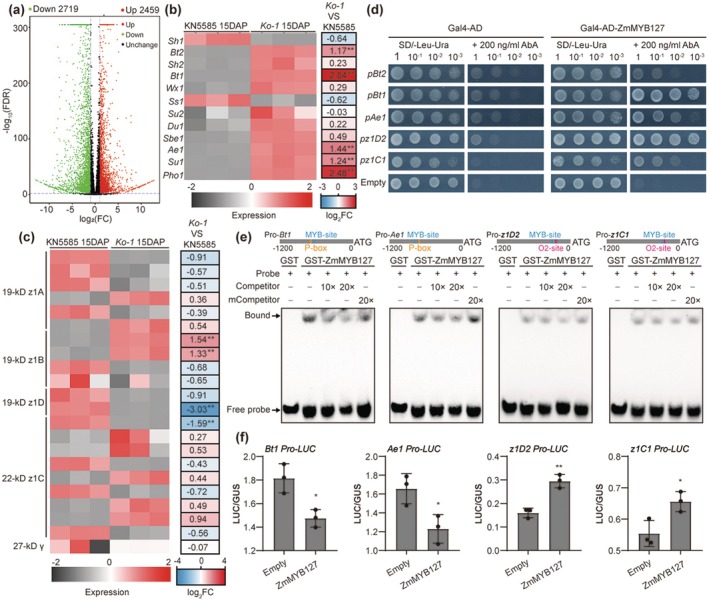
ZmMYB127 differentially regulates the expression of genes involved in starch and zein synthesis. (a) Volcano plots showing the numbers of differentially expressed genes (DEGs) between WT KN5585 and *zmmyb127* (*Ko‐1*) kernels at 15 DAP. The block dots indicate genes whose expression did not significantly change. (b) Heatmap showing the differential expression and log_2_‐fold changes of representative starch synthesis genes based on RNA‐Seq analysis. ‘**’ indicates the DEGs in *Ko‐1*. (c) Heatmap showing the differential expression and log_2_‐fold changes in the expression of the *19‐kD α‐zein*, *22‐kD α‐zein* (*z1C*) and *27‐kD γ‐zein* genes based on RNA‐Seq analysis. (d) Analysis of the interaction between ZmMYB127 and the promoters of *Bt2*, *Bt1*, *Ae1*, *z1D2 and z1C1* via yeast one‐hybrid assays. (e) EMSA showing the direct binding of ZmMYB127‐GST to the promoters of *Bt1*, *Ae1*, *z1D2 and z1C1*. (f) Effects of *ZmMYB127* transient expression on the promoter activities of *Bt1*, *Ae1*, *z1D2* and *z1C1* genes via particle bombardment of the maize endosperm at 10 DAP. The LUC/GUS ratio represents the relative activity of target promoters. Data are presented as the mean ± SD (*n* = 3). Statistical significance (*p < 0.05; **p < 0.01) was determined by two‐tailed Student's *t*‐test.

To further elucidate the regulatory effects of ZmMYB127 on kernel texture, we conducted transcriptomic deep sequencing (RNA‐Seq) on *Ko‐1* and WT KN5585 kernels at 15 DAP in three independent experiments (Figure S11). We identified 5178 annotated differentially expressed genes (DEGs) between the *Ko‐1* and WT kernels (*p* value < 0.01, absolute fold change > 2.0), with 2459 genes upregulated and 2719 downregulated (Figure 4a and Table S5).

Gene ontology (GO) and Kyoto Encyclopedia of Genes and Genomes (KEGG) enrichment analyses highlighted significant associations with nutrient reservoir activity, starch and sucrose metabolism, glycolysis and synthesis of amino acids (Figure S12). Notably, the expression of core SSGs, including *Bt2*, *Bt1*, *Ae1*, *Su1* and *Pho1*, was significantly increased in *Ko‐1* kernels (Figure 4b). Further RT‐qPCR analysis confirmed their expression patterns and revealed an overall correlation between the two datasets (Figure S13a). On the other hand, 19‐ and 22‐kD α‐zeins, encoded by a large multigene family, account for 60%–70% of the total zein fraction (Thompson and Larkins 1994; Yang et al. 2016), and 27‐kD γ‐zein is crucial for zein PB and VE formation (Liu et al. 2016; Ning et al. 2023). We analysed the expression of *19‐kD α‐zein*, *22‐kD α‐zein* and *27‐kD γ‐zein* genes in the endosperm, finding divergent changes, with a general downregulation in highly expressed genes, particularly *19‐kD α‐zeins* (*z1D*) (Figure 4c). RT‐qPCR further confirmed that transcript levels of *19‐kD* (*z1A, z1B, z1D*) and *22‐kD* (*z1C*) α‐zeins were reduced to varying degrees in *Ko‐1*, with *19‐kD z1D* almost undetectable, and *27‐kD γ‐zein* remaining unchanged (Figure S13b). Consistent with these findings, *Ko‐1* endosperm showed more SGs and fewer PBs than WT (Figure S14). Collectively, these results indicate that the *zmmyb127* mutation leads to significant alterations in storage protein and starch synthesis in the endosperm.

To explore the potential direct targets of ZmMYB127, we compared the DEGs with previously reported genes involved in its gene regulatory network (GRN) (Figure S15a) (Xiong et al. 2017). This analysis identified 69 potential target genes, including 35 upregulated and 34 downregulated DEGs (Figure S15b). Notably, three core SSGs (*Bt2*, *Bt1* and *Ae1*) and two major zein genes (*z1D2* and *z1C1*) were identified as potential direct targets of ZmMYB127 (Figure S15b). GO and KEGG analyses of these target genes revealed significant enrichment in pathways related to nutrient reservoir activity and starch and sucrose metabolism (Figure S15c,d). Promoter sequence analysis indicated several potential MYB‐binding motifs within the 1.2‐kb promoters of core SSGs and major zein genes (Figure S16). To confirm direct regulation, we first performed the yeast one‐hybrid (Y1H) assays. ZmMYB127 was found to directly bind to the promoters of *Bt1*, *Ae1*, *z1D2* and *z1C1*, but not to *Bt2* (Figure 4d). Consistently, electrophoretic mobility shift assays (EMSA) demonstrated specific binding of ZmMYB127 to the MYB‐binding sites within *Bt1*, *Ae1*, *z1D2* and *z1C1* promoters. Increasing amounts of an unlabeled WT probe markedly abolished ZmMYB127 binding to the biotin‐labelled probe, whereas the mutated unlabeled probe failed to compete for binding (Figure 4e). Moreover, transient expression assays using both particle bombardment of maize endosperm and maize leaf protoplasts further revealed that co‐expression of *Ubi‐ZmMYB127* drastically reduced the LUC activity associated with the *Bt1* and *Ae1* promoters, while significantly enhanced the LUC activity linked to the *z1D2* and *z1C1* promoters (Figure 4f and Figure S17).

### 2.5 ZmMYB127 Interacts With PBF1 and O2 to Regulate Downstream Genes

**FIGURE 5:**
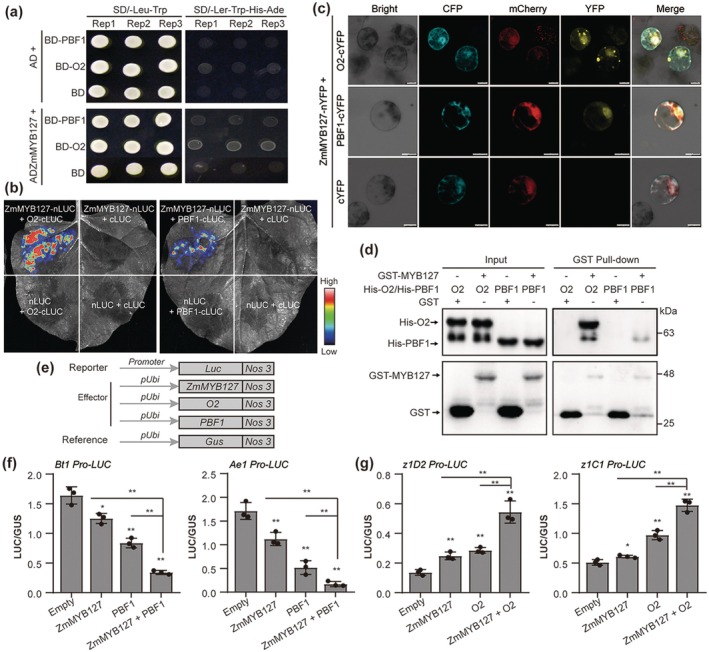
ZmMYB127 interacts with PBF1 and O2 to synergistically regulate core SSGs and zein genes, respectively. (a) ZmMYB127 interacts with PBF1 and O2 in yeast. (b, c) Split‐luciferase complementation assays (b) and bimolecular fluorescence complementation (BiFC) assays (c, Bar = 10 μm) showing the interaction of ZmMYB127 with PBF1 and O2 in maize leaf protoplasts and *Nicotiana benthamiana* leaves, respectively. (d) GST pull‐down assay showing the interaction between ZmMYB127 with O2 and PBF1. (e) Schematic diagrams of the transient expression vector constructs. (f) Quantitative transactivation of the *Bt1* and *Ae1* promoters fused with *Luc* by ZmMYB127 and PBF1. (g) Quantitative transactivation of the *z1D2* and *z1C1* promoters fused with *Luc* by ZmMYB127 and O2. The reporter plasmids with or without effector plasmids were co‐bombarded with the reference plasmids into 10‐DAP maize endosperms. As shown in (f, g), data are presented as the mean ± SD (*n* = 3). Statistical significance (*p < 0.0*5*; **p < 0.01) was determined by a two‐tailed Student's *t* test.

ZmMYB127 promoted the transcription of some major zein genes in the endosperm but lacked transactivation capacity, suggesting that it must interact with or form a complex with other regulator(s) to coordinate gene expression. To identify the potential coregulator(s), we hypothesised that they may share a similar expression pattern with *ZmMYB127*, which is highly expressed during the endosperm filling stage. We selected 30 TFs with similar endosperm filling co‐expression modules according to previous studies (Chen et al. 2014; Xiong et al. 2017), and found that O2, a well‐known endosperm‐specific TF, emerged as the best candidate due to its nearly identical expression pattern (Figure S18). Considering the interplay between O2 and PBF1 in regulating SSGs and zein genes, we also examined PBF1 as a potential ZmMYB127 interactor. We subsequently performed a yeast two‐hybrid assay, and the results revealed that the yeast cells co‐transformed with ZmMYB127 and O2 or PBF1 grew better than the corresponding negative control (empty vector) did, suggesting that ZmMYB127 can interact not only with O2 but also with PBF1 in yeast (Figure 5a).

To confirm the interaction between ZmMYB127 and O2 or PBF1, we performed a split‐luciferase complementation assay in *Nicotiana benthamiana* leaves. The results indicated that co‐infiltration with ZmMYB127‐nLUC and either O2‐cLUC or PBF1‐cLUC resulted in strong LUC activity, while such activity was absent in other co‐infiltrations (Figure 5b). Additionally, we performed a bimolecular fluorescence complementation (BiFC) assay in maize leaf protoplasts, capturing strong yellow fluorescence signals in the nucleus upon co‐expression of ZmMYB127 with either O2 or PBF1, while no fluorescence was detected in the control experiments (Figure 5c). Furthermore, we carried out a GST pull‐down assay, which demonstrated that ZmMYB127 directly associates with both O2 and PBF1 (Figure 5d). These results confirm that ZmMYB127 interacts with O2 and PBF1 in vivo and in vitro.

Previous studies have revealed that O2 and PBF1 act as key hubs in the regulatory network governing storage protein and starch synthesis in maize endosperm (Ning et al. 2023; Zhang et al. 2015, 2016). To investigate whether ZmMYB127, O2 and PBF1 co‐modulate the gene regulatory network (GRN) in the endosperm, we compared the genes regulated by ZmMYB127, O2 and PBF1 according to previous studies (Ning et al. 2023; Zhan et al. 2018). While only 27 genes were simultaneously regulated by all TFs, 314 genes were co‐regulated by ZmMYB127 and O2, accounting for 16.9% of O2‐regulated genes and 261 genes were co‐regulated by ZmMYB127 and PBF1, comprising 30.4% of PBF1‐regulated genes (Figure S19). Moreover, as expected, a MYB‐binding site adjacent to P‐box or O2‐site elements is present in the promoters of core SSGs and major zein genes, and this tandem arrangement is proximal to their transcription start sites (Figure S16). These results indicated that ZmMYB127, O2 and PBF1 coordinate to regulate target genes. To confirm this hypothesis, we performed transient expression analysis with the effector and reporter plasmids to quantify the additive effects of ZmMYB127 with PBF1 or O2 on the promoter activities of target genes (Figure 5e). The combined inhibitory effect of ZmMYB127 and PBF1 on *Bt1* and *Ae1* promoter activities was significantly greater than the individual repressive effects of either TF alone (Figure 5f). Furthermore, co‐expression of ZmMYB127 and O2 resulted in significantly stronger transactivation of *z1D2* and *z1C1* compared to their individual expressions (Figure 5g).

### 2.6 ZmMYB127 Regulates Genes Involved in Endosperm IAA Synthesis

**FIGURE 6:**
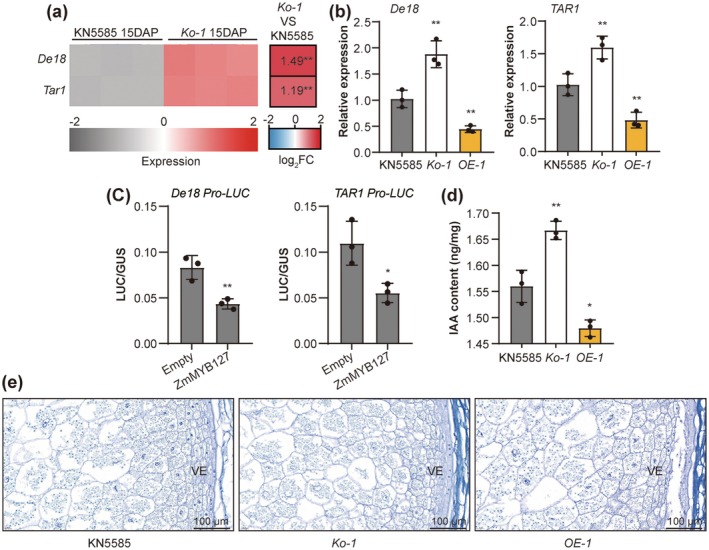
ZmMYB127 regulates IAA synthesis in the endosperm. (a) Heatmap showing the differential expression and log_2_‐fold change in the expression of major IAA synthesis genes in the endosperm based on RNA‐Seq analysis. (b) RT‐qPCR analysis of the expression of major IAA synthesis genes in the 15‐DAP kernels of the WT KN5585, *Ko‐1* and *OE‐1*. All relative expression levels were normalised to those of *Actin*. (c) Transient expression of ZmMYB127 affects the promoter activities of the *De18* and *TAR1* genes, as observed via particle bombardment of the maize endosperm at 10 DAP. (d) Determination of IAA content in WT KN5585, *Ko‐1* and *OE‐1* endosperm at 15 DAP. As shown in (b–d), data are presented as the mean ± SD (*n* = 3). Statistical significance (ns, not significant; *p < 0.05; **p < 0.01) was determined by two‐tailed Student's *t*‐test. (e) Paraffin sections of the developing endosperm of the WT KN5585, *Ko‐1* and *OE‐1* at 15 DAP. VE, vitreous endosperm.

Among the DEGs between *zmmyb127* and WT (Figure S12b), genes related to the hormone signal transduction were enriched. Moreover, tryptophan metabolism, which is involved in IAA synthesis, was enriched among the potential targets of ZmMYB127 (Figure S15d). Notably, the expression of two key IAA synthesis genes, *De18* and *TAR1* (*Tryptophan Aminotransferase Related1*), which are expressed predominantly in the endosperm, significantly increased in *Ko‐1* according to our RNA‐seq data (Figure S20a and Figure 6a). Furthermore, *ZmMYB127*, *De18* and *TAR1* are highly expressed in VE cells (Figure S20b). RT‐qPCR analysis revealed that transcript levels of *De18* and *TAR1* were significantly higher in *Ko‐1* compared to the WT KN5585, while they were lower in *OE‐1* (Figure 6b). Transient expression assay showed that LUC activity associated with the *De18* and *TAR1* promoters was significantly reduced when *Ubi‐ZmMYB127* was co‐expressed (Figure 6c). Additionally, we demonstrated that IAA remains at high levels in maize kernels during the filling phase (Figure S20c). To evaluate the impact of ZmMYB127 on IAA levels, we quantified the IAA content in the endosperm at 15 DAP. Consistent with the expression changes of IAA synthesis genes, IAA content was significantly higher in *Ko‐1* and lower in *OE‐1* compared to the WT (Figure 6d). Consequently, cytological sectioning analysis revealed that the number of VE cells in *Ko‐1* was significantly higher than that in the WT. In contrast, the *OE‐1* exhibited a lower count of VE cells compared to the WT (Figure 6e). Thus, these findings suggest that ZmMYB127 negatively regulates IAA synthesis in maize endosperm, thereby influencing endosperm development.

## 3 Discussion

**FIGURE 7:**
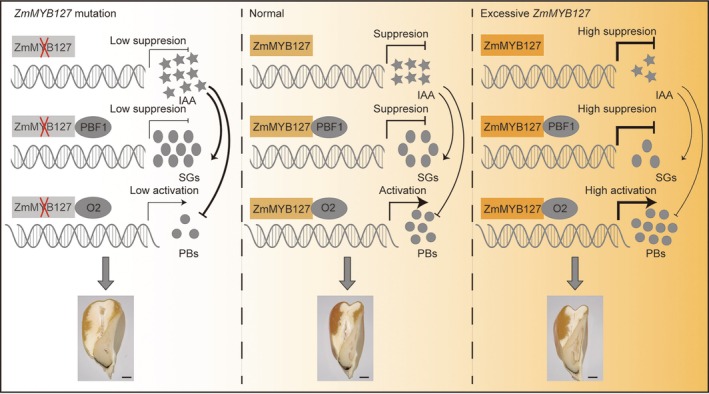
The proposed model for dual regulatory roles of ZmMYB127 in kernel texture formation during the filling stage in maize. *ZmMYB127* is specifically expressed in the endosperm and has dual functions of suppressing starch synthesis while promoting storage protein synthesis. ZmMYB127 interacts with PBF1 to additionally repress genes involved in starch synthesis and with O2 to synergistically activate genes encoding zein proteins. Thus, mutation of *ZmMYB127* alleviates the transcriptional repression of starch synthesis genes and reduces zein gene activity, resulting in increased accumulation of starch and decreased protein storage in the endosperm. Moreover, the expression of auxin synthesis genes in the endosperm is increased in *zmmyb127* mutants, leading to increased IAA content and enhanced endosperm development, thus forming larger kernels with more starchy endosperm. In contrast, excessive *ZmMYB127* increases repression of starch synthesis genes and elevates expression of zein genes, resulting in decreased starch accumulation and increased storage proteins in the endosperm. Additionally, reduced expression of auxin synthesis genes leads to lower IAA content in the excess endosperm. Together, these effects results in the formation of smaller kernels with more vitreous endosperm.

Due to excellent agronomic traits and nutritional quality of VE, it is crucial to unravel the genetic mechanisms underlying its formation in maize. Our GWAS analysis and phenotyping of transgenic kernels identified that ZmMYB127 positively regulates VE formation in maize. Furthermore, we revealed that ZmMYB127 has dual functions in regulating endosperm storage reserves: interacting with O2 to synchronously transactivate zein genes and with PBF1 to additively suppress genes involved in starch synthesis, thereby modulating VE formation. Unexpectedly, we also uncovered that ZmMYB127 negatively regulates the expression of genes involved in indole‐3‐acetic acid (IAA) synthesis in the endosperm, governing endosperm development and storage distribution. Altogether, these results indicate that ZmMYB127 modulates kernel texture and size via integrating regulation of starch, zein proteins and auxin synthesis pathways in maize endosperm (Figure 7).

### 3.1 ZmMYB127 Is a Key Regulator of Vitreous Endosperm Formation

Genetics and the environment are the main factors affecting grain texture, with the proportion of VE in maize being a quantitative trait controlled by multiple genes. While several VE‐related quantitative trait loci (QTLs) have been mapped using various maize populations, only a few genes, such as Vitreous endosperm 1 (*Ven1*) (Wang et al. 2020) and *ARF17* (Wang et al. 2024), have been functionally characterised. Substantial unexplained genetic variation related to VE remains in natural populations. Our GWAS study identified the *ZmMYB127* gene as an environmentally stable locus associated with the RVE (Figure 1). Haplotype analysis and functional studies demonstrated that ZmMYB127 positively regulates VE formation without affecting the 100‐kernel weight (Figures 1 and 2, Figures S3–S6).

The MYB TF family is one of the largest TF families in plants, divided into four subfamilies: MYB‐related, R2R3‐MYB, R1R2R3‐MYB and R4‐MYB, based on the number of adjacent DNA‐binding domain repeats (Rs) at the N‐terminus (Du et al. 2012; Martin and Paz‐Ares 1997). ZmMYB127, characterised by two adjacent Rs, is a typical R2R3‐MYB TF (Figure S8). R2R3‐MYB TFs represent the largest MYB subfamily and have been extensively studied due to their structural and functional diversity (Jiang and Rao 2020; Wu et al. 2022). The first identified plant MYB gene, *COLORED1 (C1)*, encodes an R2R3‐MYB TF required for anthocyanin synthesis in maize aleurone tissues (Paz‐Ares et al. 1987). R2R3‐MYB TFs play crucial roles in regulating secondary metabolism, influencing nutritional, medicinal, appearance and quality traits (He et al. 2020; Huang, Ming, et al. 2021). In the maize genome, 157 putative R2R3‐MYB genes have been identified (Du et al. 2012). However, their roles in kernel texture have received limited attention. A recent study identified MYB40, an R2R3‐MYB gene highly expressed in the pericarp, as a key regulator of pericarp development, resulting in a flint kernel architecture (Wang et al. 2024). Among the 157 putative R2R3‐MYB genes in maize, *ZmMYB127*, *ZmMYB73* and *ZmMYB155* are specifically expressed in the endosperm (Du et al. 2012). Recently, *ZmMYB73* was linked to the dosage‐effect defective1 (*ded1*) locus, acting as a paternal regulator of kernel set and size (Dai et al. 2022). This gene is highly expressed in the endosperm adjacent to the scutellum cell layer (EAS) during early kernel development, promoting the expression of early endosperm and EAS genes that support embryo development. Our analysis revealed that *ZmMYB127* is specifically expressed during endosperm filling and is preferentially localised in VE cells (Figure 3 and Figure S9). Remarkably, the spatiotemporal expression pattern of *ZmMYB127* closely resembles that of genes involved in zein and starch synthesis in both VE and SE cells (Zhan et al. 2015; Fu et al. 2023). Therefore, ZmMYB127 is an R2R3‐MYB TF specifically expressed in the endosperm during grain filling, promoting VE formation and contributing to kernel texture.

### 3.2 ZmMYB127 Antagonistically Regulates Starch and Zein Synthesis

The physicochemical properties of VE have been well characterised, and starch and zein synthesis during the filling stage is mechanistically related to kernel texture (Wang et al. 2020; Xu et al. 2019; Zhang and Xu 2019). Additionally, studies have revealed that higher concentrations of zeins, particularly α‐zeins, are typically found in VE rather than SE, while starch concentrations show an opposite trend between these two regions (Caballero‐Rothar 2019; Fu et al. 2023; Gayral et al. 2016; Gerde et al. 2017). Here, phenotyping and physiological analysis of the transgenic kernels demonstrated that *Ko*‐lines with less VE showed higher starch content and lower protein content (especially α‐zeins) compared to WT, while *OE*‐lines with more VE showed lower starch content and higher protein content (Figure 2). Therefore, we propose that ZmMYB127 controls VE formation by repressing starch synthesis and promoting zein protein synthesis in the endosperm. Furthermore, RNA‐Seq and biochemical analyses demonstrated that ZmMYB127 directly represses the expression of starch synthesis genes *Bt1* and *Ae1*, while transactivating *z1D2* and *z1C1* for zeins (Figure 4, Figures S15 and S17). These findings are consistent with previous studies showing that a single R2R3‐MYB TF can function as both an activator and repressor of target genes in maize and other plants (Barthole et al. 2014; Huang et al. 2020).

Starch and zeins are synthesised via distinct processes, yet TFs integrate their synthesis during endosperm filling (Chen et al. 2023; Zhang et al. 2016, 2019). The coupled synthesis of starch and storage proteins results in a trade‐off between yield and quality, with starch and storage proteins accounting for approximately 70% and 10% of kernel dry weight, respectively (Cao et al. 2024). Previous studies have highlighted that starch and zeins are coordinately synthesised during maize endosperm filling via the same TFs (Chen et al. 2023; Zhang et al. 2016, 2019). Notably, during the review of this manuscript, emerging studies reported that TaMYB44 and OsMYB44, which are orthologous to ZmMYB127 in wheat and rice, serve as negative regulators of starch synthesis in the endosperm and limit grain size and weight (Li et al. 2025; Liu et al. 2025). In this study, we demonstrate that ZmMYB127 acts as a key regulator that antagonistically modulates the allocation of storage resources by balancing starch and zein synthesis in maize endosperm. Specifically, while ZmMYB127 negatively regulates starch biosynthesis and reduces kernel size, it promotes zein accumulation and increases kernel test weight. Consequently, these opposing effects result in no net reduction in overall kernel weight (Figure 2). This dual functionality underscores the potential of ZmMYB127 as a target for breeding high‐quality maize varieties with enhanced protein content and test weight without compromising yield. The antagonistic regulation of two sets of genes prompts important questions about the underlying molecular mechanisms.

TFs interact with other proteins associated with target genes, leading to diverse outcomes in gene expression. In maize, O2 and its heterodimerization partners (OPH1/2) physically interact with PBF1, binding to the O2 site (TT/CCACGT) and P‐box (TGTAAAG), respectively, to additively transactivate target genes involved in starch and zein synthesis in the endosperm (Zhang et al. 2015, 2016). Interestingly, a recent study showed that PBF1 represses the expression of core SSGs under normal nitrogen supply, contributing to the balance of protein and carbohydrate storage in maize endosperm (Ning et al. 2023). This observation suggests that the O2 and PBF1 transcriptional regulatory network coordinates starch and storage protein synthesis in the endosperm. The similar expression patterns of *ZmMYB127*, *O2* and *PBF1* indicate potential interactions among them. We have confirmed that ZmMYB127 interacts with both O2 and PBF1 in vivo and in vitro (Figure S18 and Figure 5a–d). Moreover, transient expression assays revealed that ZmMYB127 and PBF1 additively suppress the expression of *Bt1* and *Ae1*, while ZmMYB127 and O2 synergistically transactivate *z1D2* and *z1C1* expression for zein synthesis (Figure 5e–g). In rice, the core motif of the MYB binding site, AACA, is conserved in the promoters of glutelin‐encoding genes and is essential for regulating endosperm‐specific expression (Takaiwa et al. 1996). Moreover, the R2R3‐MYB TF OsMYB5 binds to the AACA motif in the promoters of glutelin genes (Suzuki et al. 1998). In maize, the MYB‐site coexists with the O2‐site or P‐box in the promoters of zein genes and core SSGs (Figure S16).

We propose that ZmMYB127 physically interacts with O2 and PBF1, binding to the MYB‐site, O2‐site and P‐box respectively, to precisely control the synthesis of zeins and starch in maize endosperm. However, further biochemical assays and comparative studies are necessary to confirm whether ZmMYB127 physically interacts with O2 and PBF1 in a complex to synergistically regulate the expression of zein genes and SSGs.

### 3.3 ZmMYB127 Regulates Endosperm Development Partly Through Affecting IAA Synthesis

A recent study revealed that TaMYB44 in wheat, an ortholog of ZmMYB127, also restricts grain size by modulating secondary metabolism and hormone homeostasis (Liu et al. 2025). In cereal endosperm, IAA plays a crucial role in regulating cell division, development and organ formation (Doll et al. 2017; Locascio et al. 2014). The high‐level, spatial and temporal expression of IAA synthesis genes in the endosperm indicates that these genes are regulated primarily at the transcriptional level (Figure S20). However, specific regulators controlling IAA synthesis in maize endosperm remain limited (Song et al. 2024; Zhang et al. 2022). While several studies have linked R2R3‐MYB TFs to IAA metabolism and signalling (Ke et al. 2021; Wang et al. 2024), few have demonstrated that these TFs directly regulate IAA synthesis in maize endosperm. In this study, we showed that ZmMYB127 negatively regulates the expression of *De18* and *TAR1*, both involved in IAA synthesis, resulting in increased IAA content in the *Ko‐1* and decreased in the *OE‐1* (Figure 6). Furthermore, our study indicates a clear increase in total cell number in the *Ko‐1* endosperm, correlating with larger kernel size, while the *OE‐1* kernels are smaller and have fewer endosperm cells (Figure 6e and Figure 2). This suggests that ZmMYB127 may regulate IAA synthesis to modulate endosperm cell number, thereby affecting kernel size in maize.

A deficiency in IAA impairs carbon and nitrogen allocation in maize endosperm, with IAA directly or indirectly regulating the transcription of SSGs and zein genes (Bernardi et al. 2019). Interestingly, the *de18* endosperms show reduced starch content and expression of core SSGs (including *Sh1*, *Bt2*, *Sh2* and *Ae*), while the expression of zein genes and nutritional value is higher than those in WT endosperms (Bernardi et al. 2019). Therefore, we speculate that ZmMYB127 has a dual effect on starch and zein synthesis in the endosperm. Beyond its direct antagonism of the regulation of starch and protein synthesis, ZmMYB127 also negatively regulates auxin synthesis, which in turn influences starch and protein components. A recent study highlights a hierarchical regulatory network centred on ZmNAC128/130 that coordinates storage reserves and IAA synthesis, indicating an interplay between these processes in maize endosperm (Song et al. 2024). Our study introduces another regulatory network centred on ZmMYB127, which integrates storage reserves and IAA synthesis in maize endosperm. However, the mechanisms by which ZmMYB127 affects these pathways warrant further investigation.

In summary, our work identified ZmMYB127, an endosperm‐specific R2R3‐MYB TF associated with the RVE in maize, and revealed that ZmMYB127 constitutes a new regulatory mechanism that modulates kernel texture and size by integrating multiple regulatory pathways. Moreover, ZmMYB127 has potential applications in molecular breeding for kernel quality through marker‐assisted selection, as well as in manipulating kernel architecture to meet specific agricultural needs.

## 4 Materials and Method

### 4.1 Plant Materials and Growth Conditions

The population used for the genome‐wide association study (GWAS) analysis consisted of 238 maize inbred lines (Table S1). Detailed information on all the inbred lines in the association panel can be found in our previous study (Yang et al. 2024). These inbred lines were planted in Chongzhou, Sichuan Province, in 2019 (2019CZ), Xinxiang, Henan Province, in 2019 (2019XX), and Chongzhou, Sichuan Province, in 2020 (2020CZ). The field trials were conducted in a randomised complete block design with two replicates per environment. Each inbred line was planted in two‐row plots with a row length of 3 m, a row spacing of 0.75 m and 14 plants per row. Standard maize cultivation practices were followed, and manual self‐pollination was performed. The kernels were harvested after full maturity.

The maize tissues and kernels were collected from the maize inbred line B73. Different tissues (root, shoot, leaf, silk, anthers, pollen, tassel, cob, seed, endosperm, embryo) and the developing seeds at 3, 6, 9, 12, 15, 18, 21, 24, 27, 30, 33 and 36 days after pollination (DAP) were collected from the maize inbred line B73 to determine the specific expression pattern of *ZmMYB127*. The materials were immediately frozen in liquid nitrogen and stored at −80°C until use for RNA extraction. Samples were collected from three individual plants for each developmental stage.

For CRISPR‐Cas9 knockout mutants (in the KN5585 and B104 background), the specific gRNA of *ZmMYB127* was designed by CRISPR‐P v2.0 (http://crispr.hzau.edu.cn/cgi‐bin/CRISPR2/CRISPR), according to the high similarity in gene sequences. For the overexpression lines (in the KN5585 background), the full length of the coding sequence (CDS) of *ZmMYB127* was cloned into the pCAMBIA3301 vector and driven by the *Ubiquitin* promoter. All transgenic lines were created through *Agrobacterium tumefaciens* ‐mediated transformation. All transgenic materials were cultivated on the experimental field at Sichuan Agricultural University in Sichuan, China and in Sanya, China.

### 4.2 Measurement of the Ratio of Vitreous Endosperm

Mature maize kernels were longitudinally cut in the middle of the embryos, then the VideometerLab UV multi‐spectral imaging system (Videometer A/S, Hørkær 12B, DK‐2730 Herlev) was used to collect longitudinal section images of maize kernels. The images were captured using the VideometerLab 3.6.9 software (Videometer A/S), the areas of the endosperm and the floury endosperm of the kernels were extracted, and the proportion of VE was calculated. Six well‐pollinated and generally representative ears were selected, and then ten kernels from the middle region of the ear were collected for measurement.

### 4.3 Genome‐Wide Association Analysis (GWAS)

All samples in the association panel were genome‐wide resequenced in the previous study, and a total of 2,431,295 high‐quality SNPs were obtained (Yang et al. 2024). Phenotypic values were taken as the mean of RVE for all inbred lines in each environment. The FarmCPU model in the rMVP package (version 1.0.6) was used for GWAS, and significance in marker‐trait associations was determined by the Bonferroni correction threshold (*p* = 1/N, where N is the number of genome‐wide markers, *p* < 4.11 × 10^−7^). Significant SNPs identified in at least two environments were referred to as co‐located SNPs, and the linkage disequilibrium (LD) decay distance (10 kb) around the co‐located SNPs was considered as a QTL. Candidate genes within the QTL interval were functionally annotated by referring to the B73_V4 genome, (https://www.maizegdb.org/gbrowse).

The BLUE value of RVE was used to analyse the association between candidate genes and SNPs within 2000 bp upstream and downstream of the CDS. For the candidate gene association analysis, the general linear model provided by TASSEL 5.0 (Bradbury et al. 2007) was applied. The significance threshold was set at *p* = 0.05/*N* (*N* = 69, *p* = 7.25 × 10^−4^), where N represents the number of markers within the specified region. Haplotypes were defined based on the allele types of significant SNPs. Student's *t*‐test was then performed to assess phenotypic differences between these haplotypes, allowing the identification of the most favourable haplotypes.

### 4.4 Genotyping

To identify the transgenic materials, leaves were harvested and the genomic DNA was extracted using the cetyl trimethylammonium bromide (CTAB) method. For CRISPR‐Cas9 knockout mutants, the Cas9 cassette was detected by PCR identification with specific primers listed in Table S6. The Cas9‐target sites were amplified and analysed by sequencing. For the overexpression materials, the sequences of *ZmMYB127* were amplified from genomic DNA using specific primers to genotype the transgenic materials. The overexpression level was analysed by RT‐qPCR; the primers are listed in Table S6.

### 4.5 Physiological Analyses of Maize Kernels

Well‐pollinated ears in the middle of each row were used to evaluate 100‐kernel weight and test weight based on a seed phenotyping system (Jielaimei, Sichuan, China). Mature kernels were longitudinally cut in the middle of the embryos and imaged on a Leica stereomicroscope; then VE area was quantified using the ImageJ software. Kernel length, width and thickness were scored using a vernier calliper.

Mature kernels were ground into fine powders to quantify the levels of starch and protein. Starch content was measured following the manufacturer's instructions (Megazyme Total Starch Kit; KTSTA‐50A). Total amino acid (TAA) and free amino acid (FAA) contents were quantified according to the protocols that were provided (Huang et al. 2022). For the zein protein extraction, a total of 50 mg fine powder from each sample was prepared and incubated in 0.5 mL zein extraction buffer (3.75 mM sodium borate, 2% 2‐mercaptoethanol [v/v], 0.3% SDS [w/v] and 70% ethanol [v/v]; pH 10). The non‐zein protein was extracted by non‐zein extraction buffer (2.5 mM sodium borate, 2% 2‐mercaptoethanol [v/v] and 5% SDS [w/v]; pH 10). The zein and non‐zein accumulation was visualised by 15% SDS‐PAGE gel electrophoresis and quantified by using a BCA protein assay kit (Thermo Scientific; 23227). These experiments were performed according to the protocols that were provided (Huang et al. 2022).

The IAA content of developing kernels of B73, and IAA concentration in *Ko‐1*, *OE‐1* and wild type (KN5585) kernels at 15‐DAP were quantified according to the protocols that were provided (Song et al. 2024).

### 4.6 Histocytochemical Analysis

To observe endosperm texture and SGs, mature kernels were cut along the longitudinal axis and then observed under a scanning electron microscope (Zeiss Merlin Compact).

For light microscopy, the immature 15 DAP kernels of *zmmyb127* and WT were cut longitudinally cutting and fixed in FAA buffer for paraffin preparation. The sections were cut by a Leica microtome, stained with haematoxilin giemsa acid fuchsin or toluidine blue, and imaged under a bright field by a Leica microscope.

For transmission electron microscope (TEM), the immature 15 DAP kernels of *zmmyb127* and WT were sliced and fixed in phosphate buffer (pH 7.2) with 2.5% (w/v) glutaraldehyde. The sections were examined and photographed with a transmission electron microscope (Hitachi; H7600).

### 4.7 RNA Extraction and RT‐qPCR

The total RNA extraction was performed according to the protocol of the FastPure Universal Plant Total RNA Isolation Kit (Vazyme; RC411‐01), and first‐strand cDNA synthesis was performed according to the HiScript II Q RT SuperMix kit (Vazyme; R212‐02). Real‐time PCR experiments were performed on a CFX96 Real‐Time System (Bio‐Rad, USA) according to the instructions of ChamQ Universal SYBR qPCR Master Mix (Vazyme; Q711‐02). Three independent biological replicates from three different ears were performed. The relative gene expression was analysed according to the 2^−ΔΔCt^ method, and maize *β‐actin* was used as the internal reference gene. All primer details are listed in Table S6.

### 4.8 RNA‐Seq Analysis

Total RNA was extracted from 15 DAP kernels of homozygous *zmmyb127* (*Ko‐1*) and WT, which were harvested from three heterozygous ears separately. The RNA libraries were constructed and sequenced by APTBIO (Shanghai). After sequencing, raw reads were subjected to quality control by FastQC (v.0.11.9) and mapped to the reference maize genome (Zm‐B73‐Reference‐NAM‐5.0) using Hisat2 (v2.0.5). The gene expression value was estimated by FeatureCounts (v2.0.1), and the differentially expression genes (DEGs) were identified with the thresholds of a |log_2_FC (fold change)| > 1.0 and *p*‐value < 0.01. The GO enrichment profile and KEGG enrichment profile of DEGs were performed using the program Phyper (http://www.geneontology.org/) and KEGG Mapper (http://www.genome.jp/kegg/pathway.html), respectively. The heatmap was drawn by MeV 4.9.0. The gene regulatory network (GRN) was displayed by Cytoscape_3.8.0.

### 4.9 RNA In Situ Hybridization

RNA in situ hybridization was conducted on 12‐DAP B73 kernels. The cDNA fragments of ZmMYB127 were amplified, and antisense and sense RNA probes were synthesised through in vitro transcription using T7 RNA polymerase with digoxigenin RNA Labeling Mixture (Roche). Tissue processing and hybridization experiments were carried out by following previously published methods (Zhang et al. 2015).

### 4.10 Subcellular Localization

The ORF of ZmMYB127 without the stop codons was cloned and fused into the vector pCAMBIA2300. The empty vector pCAMBIA2300 was used as a control. These constructs were introduced into onion epidermal cells by particle bombardment and into maize leaf protoplasts by PEG‐Ca^2+^ mediated transfection, respectively. The green fluorescent signal was observed by the A1R‐si Laser Scanning Confocal Microscope (Nikon, Japan) under blue excitation light at 488 nm, after 48 h of growth.

### 4.11 Transcriptional Activation Analysis

The ORF of ZmMYB127 was cloned and fused with GAL4‐BD of the vector pGBKT7 (Invitrogen) expression. The empty pGBKT7 was used as a negative controls, and pGBKT7‐Opaque2 was used as the positive control. All the constructs were transformed into the yeast strain AH109 by the PEG/LiAc method. The yeast transformants were screened on SD/−Trp and SD/−Trp‐His‐Ade plates. The experiment was conducted following the provided protocol (Zhang et al. 2014). In addition, the transcriptional repression activity was assessed using a dual‐luciferase reporter assay. The coding sequence of ZmMYB127 was cloned into the GAL4‐DB vector under the control of the CaMV 35S promoter to generate an effector plasmid. The LUC driven by the *35S* mini promoter containing 5xGAL4 binding sites, and the Renilla LUC driven by the *35S* promoter served as the reference. The known activator Opaque2 and an empty vector served as positive and negative controls. These plasmids were co‐transfected into maize leaf protoplasts. The ratio of LUC to REN activity was then measured using the dual‐Luciferase assay system (Promega) according to the manufacturer's instructions.

### 4.12 Yeast One‐Hybrid (Y1H) Assay

The promoters of core starch synthetic genes and zein genes were constructed into the pABAi vector and used as the reporter vectors. The coding region of ZmMYB127 fusing to pGADT7 was used as the effector. These constructs were co‐transformed into the Y1H yeast strain. The transformed yeast (1, 1/10, 1/100 and 1/1000 culture dilutions) was grown and screened on SD/‐Leu‐Ura or SD/‐Leu‐Ura + 200 ng/mL Aureobasidin A (AbA) (Coolaber; CA2332G) medium, respectively. The experiment was conducted in accordance with the Matchmaker Gold Y1H System User Manual (Clontech).

### 4.13 Transcriptional Activity Assay

The LUC driven by the promoters of downstream target genes was used as a reporter vector. ZmMYB127 driven by the *Ubi* promoter was used as an effector vector. The transient expression vector was modified from the plant expression vector, pBI221. The GUS driven by the *Ubi* promoter was used as a reference vector. Depending on the experimental system, the vectors were either co‐transformed into maize endosperm by particle bombardment or co‐transfected into maize mesophyll protoplasts. The ratio of LUC/GUS activity was determined and measured following the protocol described by our previous reporter (Hu et al. 2012; Li et al. 2018).

### 4.14 Protein–Protein Interaction Assay

#### 4.14.1 Yeast Two‐Hybrid (Y2H) Assay

The ORFs of O2 and PBF1 were cloned and fused into the pGBKT7 vector. The ZmMYB127 was fused into the pGADT7 vector. The different combinations of the constructs were co‐transformed into the Y2H gold yeast strain. The Y2H gold yeast cells from each transformation system (1, 1/10, 1/100, 1/1000 culture dilutions) were screened on SD/‐Leu‐Trp and SD/‐Leu‐Trp‐His‐Ade plates. The experiment was performed according to the user manual of the MatchMaker Gold Y2H system.

#### 4.14.2 Split Luciferase Complementation Assay

The ORFs of O2 and PBF1 were fused into cLUC, while ZmMYB127 into nLUC. Transformation of *N. benthamiana* leaf epidermal cells were injected into *N. benthamiana* leaves. The Luc images were obtained after 2 days of cultivation by a ChemiDoc system (BioRad). The experiment was carried out in accordance with the protocol given in the paper (Zhao et al. 2018).

#### 4.14.3 Bimolecular Fluorescence Complementation (BiFC) Assay

The coding regions of O2 and PBF1 were fused to a pRTVcVC vector, while ZmMYB127 was cloned into a pRTVcVN vector. Equal amounts of the combined plasmids were transformed into maize leaf protoplasts. The YFP signal was observed using a scanning confocal microscope (Leica, STELLARIS STED/EM CPD300) after 24 h dark incubation.

#### 4.14.4 Pull‐Down Assay

The coding sequence of ZmMYB127 was inserted into plasmid pGEX‐6 T to generate a GST fusion construct. Recombinant proteins GST‐ZmMYB127 and GST (as control) were expressed in *E. coli* Rosetta (DE3) cells and affinity‐purified using GST‐tag Purification Resin (Beyotime; P2251). Separately, the coding sequences of PBF1 and Opaque2 were cloned into pET32a to produce His‐tagged fusion proteins. His‐PBF1 and His‐O2 were similarly expressed in *E. coli* and purified using His‐tag Purification Resin (Beyotime; P2233). For pull‐down assays, purified GST‐ZmMYB127 or GST control were immobilised onto Glutathione Sepharose beads at 4°C for 2 h. Equal amounts of His‐PBF1 or His‐O2 were then added to the beads and incubated for another 2 h. After washing five times with PBS buffer, bound proteins were eluted in SDS loading buffer, resolved by 10% SDS‐PAGE, and analysed by immunoblotting using anti‐GST (ABclonal; AE077) and anti‐His antibodies (Beyotime; AF5060).

### 4.15 Electrophoretic Mobility Shift Assay (EMSA)

Oligonucleotide probes were synthesised and end‐labelled by Sangon Biotech. Recombinant GST‐ZmMYB127 protein was expressed in *E. coli* Rosetta (DE3) cells and affinity‐purified with GST‐tag Purification Resin (Beyotime; P2251). The EMSA assays were carried out following the protocol of LightShift Chemiluminescent EMSA kit (Thermo Scientific). Briefly, the purified protein was incubated with a biotin‐labelled probe at 25°C for 30 min. The reaction mixtures were then separated on a native‐PAGE gel, and the protein‐DNA complexes were visualised using the SuperSignal West Femto (Thermo Scientific, 34 095).

### 4.16 Statistical Methods

The results from each group are shown as the mean ± SD (standard deviation) from at least three independent experiments. All bar charts were generated by GraphPad Prism 8. Microsoft Excel (2019) was used to calculate *p*‐value by paired two‐sided Student's *t* test methods (*p < 0.05; **p < 0.01).

## Author Contributions

Conceptualization: T.L., J.W. and Y.L.; methodology: Y.W. (Yayun Wang), Z.L., Y.W. (Yongbin Wang), C.M., D.W., A.Q., Q.L. and J.Y.; Investigation: T.L., Y.L., L.G., X.F. and Y.H. (Yufeng Hu); writing – original draft: Y.L. and T.L.; writing – review and editing: T.L., Y.W. (Yayun Wang), Y.H. (Yubi Huang); project administration: T.L. and Y.H. (Yubi Huang).

## Disclosure

Accession Numbers: Sequence data from this article can be found in the MaizeGDB (https://maizegdb.org) database under accession number.

## Conflicts of Interest

The authors declare no conflicts of interest.

## Supporting information


**Figure S1:** Measurement of the ratio of vitreous endosperm (RVE) in longitudinal section of mature kernels by threshold segmentation.
**Figure S2:** GWAS analysis of the RVE in maize kernels.
**Figure S3:** Comparison of the 100‐kernel weight and ZmMYB127 relative expression between HAP1 and HAP2 lines.
**Figure S4:** Kernel phenotypes of maize zmmyb127 knock‐out lines in the KN5585 background.
**Figure S5:** Kernel phenotypes of ZmMYB127‐overexpression lines in the KN5585 background.
**Figure S6:** Kernel phenotypes of maize zmmyb127 knock‐out line in the B104 background.
**Figure S7:** Measurement and comparison of total amino acid (TAA) and free amino acid (FAA) in WT and zmmyb127 mature kernels.
**Figure S8:** The primary sequence and phylogenetic analysis of ZmMYB127.
**Figure S9:** Expression pattern of ZmMYB127.
**Figure S10:** The functional characters of transcription factor ZmMYB127.
**Figure S11:** Characteristics of the RNA‐Seq analysis.
**Figure S12:** GO and KEGG enriched analysis of DEGs in Ko‐1 developing kernels versus WT at 15‐DAP.
**Figure S13:** RT‐qPCR analysis of starch synthesis genes and zein gene expression in the 15‐DAP kernels of WT KN5585 and Ko‐1.
**Figure S14:** Light microscopy observations and transmission electron microscopy images of 15‐DAP endosperm of the WT and zmmyb127 at the starchy endosperm (SE).
**Figure S15:** Analysis of potential target genes of ZmMYB127.
**Figure S16:** Analysis of MYB‐binding sites, PBF1‐binding sites and O2‐binding sites in the promoters of core SSGs, major zein genes and IAA synthesis genes.
**Figure S17:** Effect of ZmMYB127 overexpression on the promoter activity of Su1, z1A1 and z1B4 genes via particle bombardment of maize endosperm at 10‐DAP.
**Figure S18:** Expression pattern relationships between ZmMYB127, PBF1 and O2.
**Figure S19:** Venn diagram showing the relationships between ZmMYB127 regulated genes, PBF1 regulated genes and O2 regulated genes.
**Figure S20:** IAA synthesis in maize kernel.

**Table S1:** The ratio of vitreous endosperm of 238 inbred maize lines in three different environments.

**Table S2:** Statistics the substantial variation of the RVE in three different environments.

**Table S3:** GWAS identified 14 significant loci and corresponding to 29 candidate genes associated with RVE.

**Table S4:** List of haplotypes of 238 maize inbred lines based on the three most significant SNPs associated with genetic RVE.

**Table S5:** Genes differentially expressed in zmmyb127 (Ko‐1) kernels compared with WT kernels at 15‐DAP.

**Table S6:** Primers used in this study.

## Data Availability

The raw data of RNA‐Seq analysis generated for this study has been submitted to the National Center for Biotechnology Information (NCBI) database under accession numbers PRJNA1172606.
