# Supplementary material for: ZmMYB127 Modulates Maize Kernel Texture and Size by Integrating the Synthesis of Starch, Zein Proteins and Auxin
Source: Plant Biotechnol J. 2025 Sep 24;24(2):810–27. doi: 10.1111/pbi.70384 (PMC12906810; doi:10.1111/pbi.70384)
Supplement: Supplementary file 1 — Figure S1: Measurement of the ratio of vitreous endosperm (RVE) in longitudinal section of mature kernels by threshold segmentation. Figure S2: GWAS analysis of the RVE in maize kernels. Figure S3: Comparison of the 100‐kernel weight and ZmMYB127 relative expression between HAP1 and HAP2 lines. Figure S4: Kernel phenotypes of maize zmmyb127 knock‐out lines in the KN5585 background. Figure S5: Kernel phenotypes of ZmMYB127‐overexpression lines in the KN5585 background. Figure S6: Kernel phenotypes of maize zmmyb127 knock‐out line in the B104 background. Figure S7: Measurement and comparison of total amino acid (TAA) and free amino acid (FAA) in WT and zmmyb127 mature kernels. Figure S8: The primary sequence and phylogenetic analysis of ZmMYB127. Figure S9: Expression pattern of ZmMYB127. Figure S10: The functional characters of transcription factor ZmMYB127. Figure S11: Characteristics of the RNA‐Seq analysis. Figure S12: GO and KEGG enriched analysis of DEGs in Ko‐1 developing kernels versus WT at 15‐DAP. Figure S13: RT‐qPCR analysis of starch synthesis genes and zein gene expression in the 15‐DAP kernels of WT KN5585 and Ko‐1. Figure S14: Light microscopy observations and transmission electron microscopy images of 15‐DAP endosperm of the WT and zmmyb127 at the starchy endosperm (SE). Figure S15: Analysis of potential target genes of ZmMYB127. Figure S16: Analysis of MYB‐binding sites, PBF1‐binding sites and O2‐binding sites in the promoters of core SSGs, major zein genes and IAA synthesis genes. Figure S17: Effect of ZmMYB127 overexpression on the promoter activity of Su1, z1A1 and z1B4 genes via particle bombardment of maize endosperm at 10‐DAP. Figure S18: Expression pattern relationships between ZmMYB127, PBF1 and O2. Figure S19: Venn diagram showing the relationships between ZmMYB127 regulated genes, PBF1 regulated genes and O2 regulated genes. Figure S20: IAA synthesis in maize kernel. [file PBI-24-810-s004.docx]

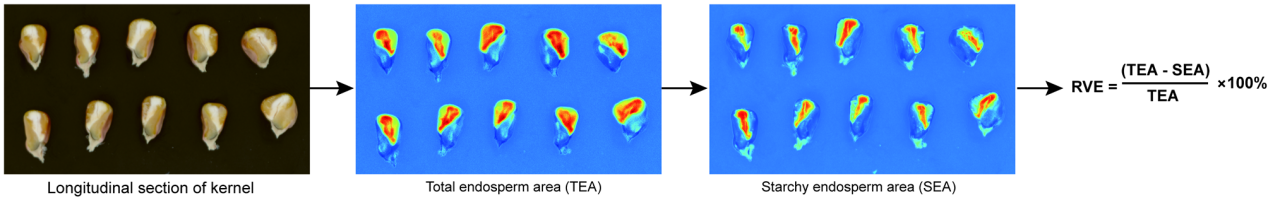


**Supplemental Figure 1. Measurement of the ratio of vitreous endosperm (RVE) in longitudinal section of mature kernels by threshold segmentation.**


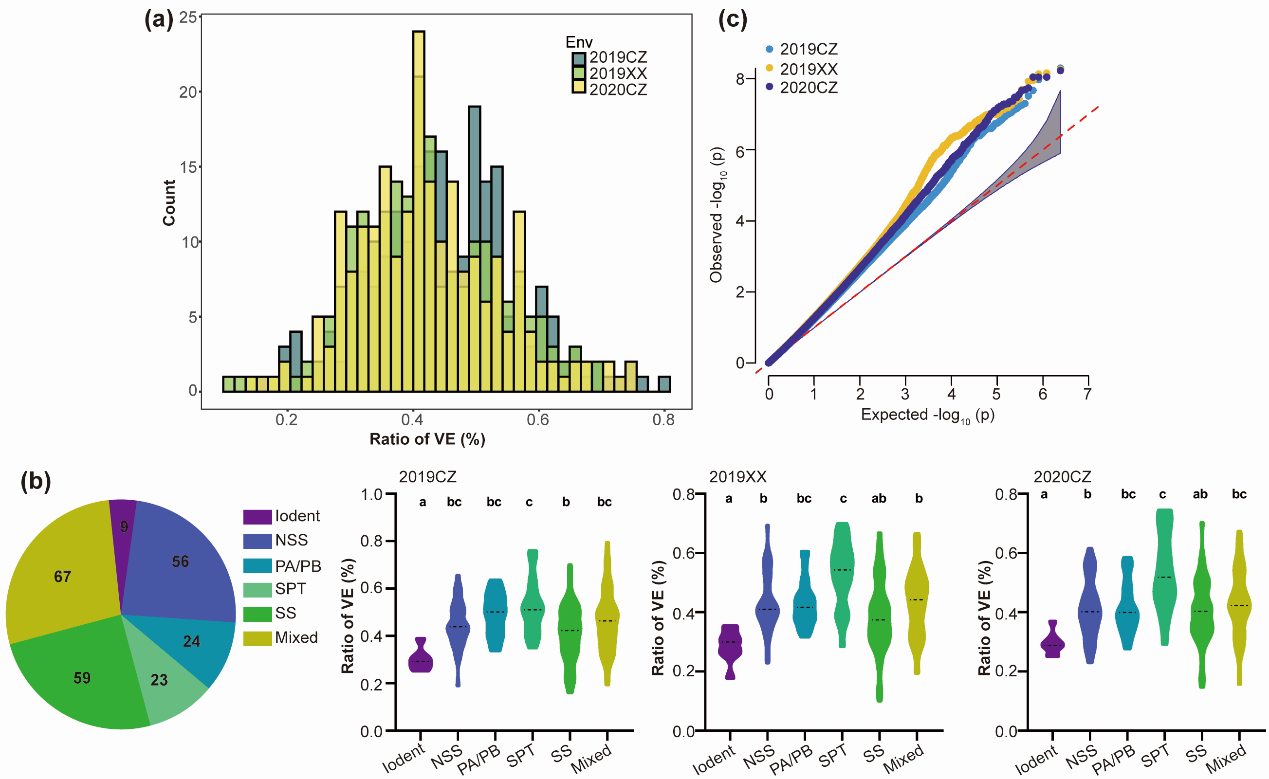


**Supplemental Figure 2. GWAS analysis of the RVE in maize kernels**.

(a) Distribution of the RVE in longitudinal section of mature kernels from 238 inbred lines using OriginPro software. (b) Kernel texture of 6 subpopulations revealed by RVE. Iodent, Iowa experiment station reid yellow dent; NSS, Non-stiff stalk; PA/PB, Group A/Group B germplasm derived from modern U.S. hybrids; SPT, Si ping tou; SS, Stiff stalk; Mixed, lines of mixed origin. (c) Quantile–quantile plot for GWAS under a FarmCPU model.


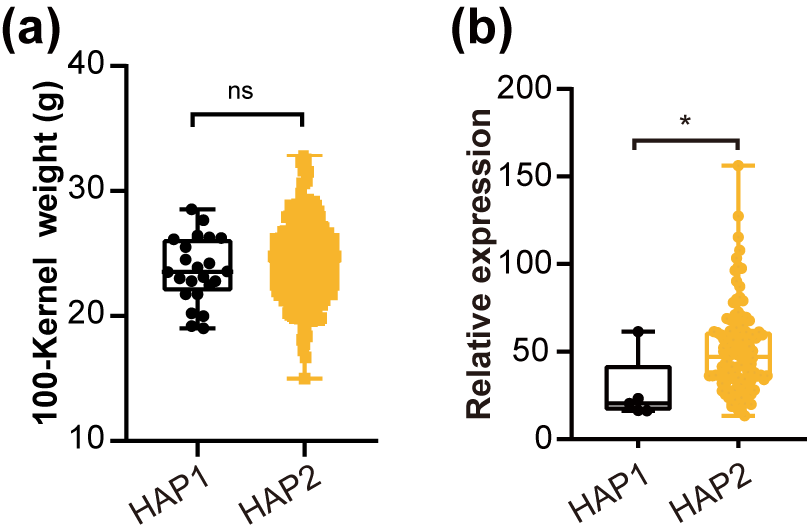


**Supplemental Figure 3. Comparison of the 100-kernel weight and *ZmMYB127* relative expression between HAP1 and HAP2 lines.**

(a) Comparison of the 100-kernel weight between HAP1 and HAP2 lines. The boxplot showing the 100-kernel weight of Hap1 (GTC, n=24) and Hap2 (ACG, n=210). The best linear unbiased estimate (BLUE) values of 100-kernel weight from three different environments were used for analysis. (b) Boxplot showing ZmMYB127 relative expression among 121 diverse inbred lines of Hap1 (n=5) and Hap2 (n=116). Data are presented as means ± s.d. Statistical significance (ns, not significant; **P* < 0.05) was determined by a two-tailed Student’s *t* test, as shown in (a,b).


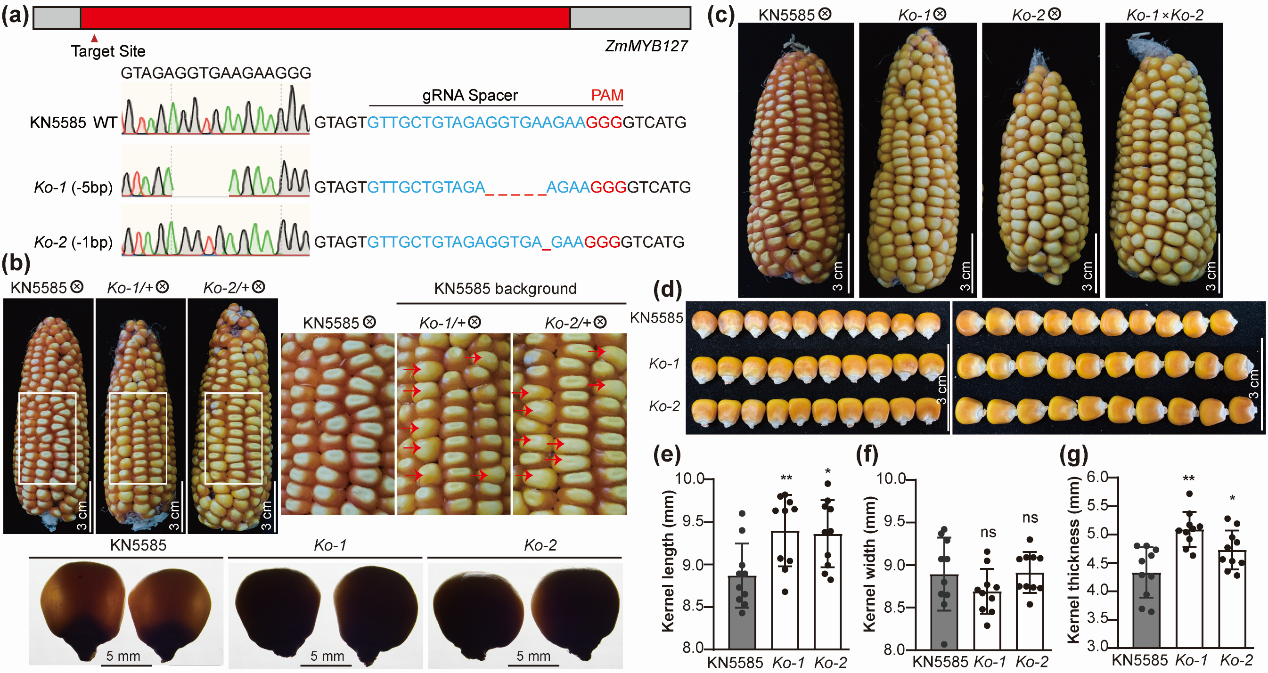


**Supplemental Figure 4. Kernel phenotypes of maize *zmmyb127* knock-out lines in the KN5585 background.**

1. Schematic diagram of the CRISPR/Cas9-edited sequences in *zmmyb127* alleles. The arrow indicates the guide RNA (gRNA) target site. Two independent knockout lines were recovered with 5 bp deletion for *KO-1* and 1bp deletion for *KO-2* in the KN5585 inbred line. The gRNA spacer and the protospacer adjacent motif (PAM) site are indicates. (b) Ear and kernel phenotypes of the wild-type (WT) KN5585 and *zmmyb127*. Upper panel, self-pollinated WT KN5585, *Ko-1*/+, and *Ko-2*/+. The red arrowheads indicate the *zmmyb127* mutant kernel. Lower panel, WT KN5585 and *zmmyb127* mature kernels observed in a light box. (c) Allelic test of *Ko-1* and *Ko-2* alleles. The phenotypes of self-pollinated KN5585, *Ko-1*, *Ko-2*, and the cross between *Ko-1* and *Ko-2*. (d) Comparisons of the kernel length and width between WT KN5585 and *zmmyb127* mature kernels. (e-g) Measurement of kernel length (e), kernel width (f) and kernel thickness (g) between WT KN5585 and *zmmyb127* mature kernels. Data are presented as means ± s.d. Statistical significance (**P* < 0.05; ***P* < 0.01) was determined by two-tailed Student’s *t*-test.


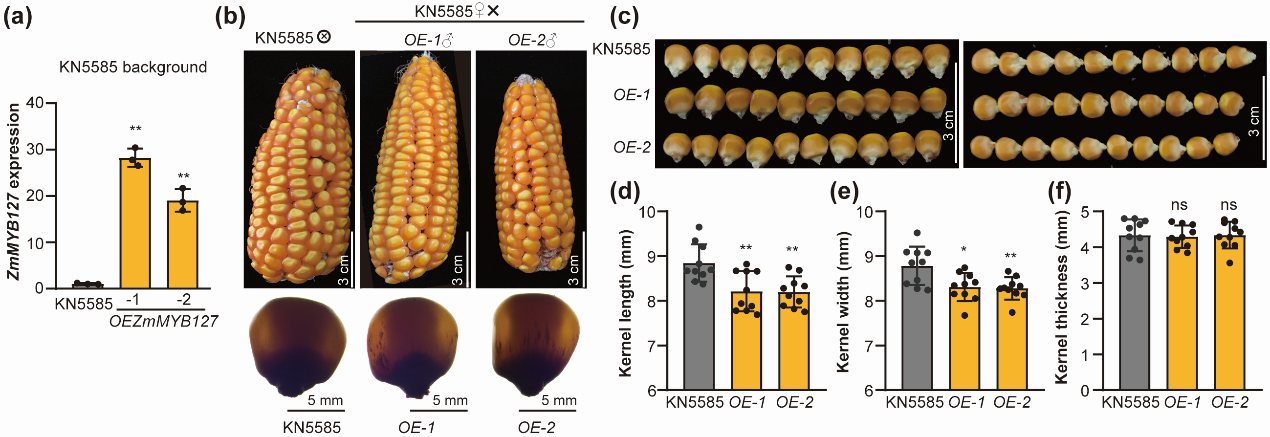


**Supplemental Figure 5. Kernel phenotypes of *ZmMYB127*-overexpression lines in the KN5585 background.**

1. RT-qPCR analysis of *ZmMYB127* expression in the WT KN5585 and *ZmMYB127*-overexpressing lines (-1 and -2) in the KN5585 background at 15-DAP kernels. Data are presented as means ± s.d. (n=3). (b) Ear and kernel phenotypes of the WT KN5585 and *ZmMYB127*-overexpressing. Upper panel, self-pollinated WT KN5585, and the cross between WT KN5585 and *ZmMYB127*-overexpressing lines. Lower panel, WT KN5585 and *ZmMYB127*-overexpressing mature kernels observed in a light box. (c) Comparisons of the kernel length and width between WT KN5585 and *ZmMYB127*-overexpressing mature kernels. (d-f) Measurements of kernel length (d), kernel width (e) and kernel thickness (f) between WT KN5585 and *ZmMYB127*-overexpressing mature kernels. Data are presented as means ± s.d. (n=10). Statistical significance (ns, not significant; **P* < 0.05; ***P* < 0.01) was determined by two-tailed Student’s *t*-test, as shown in (a,d-f).


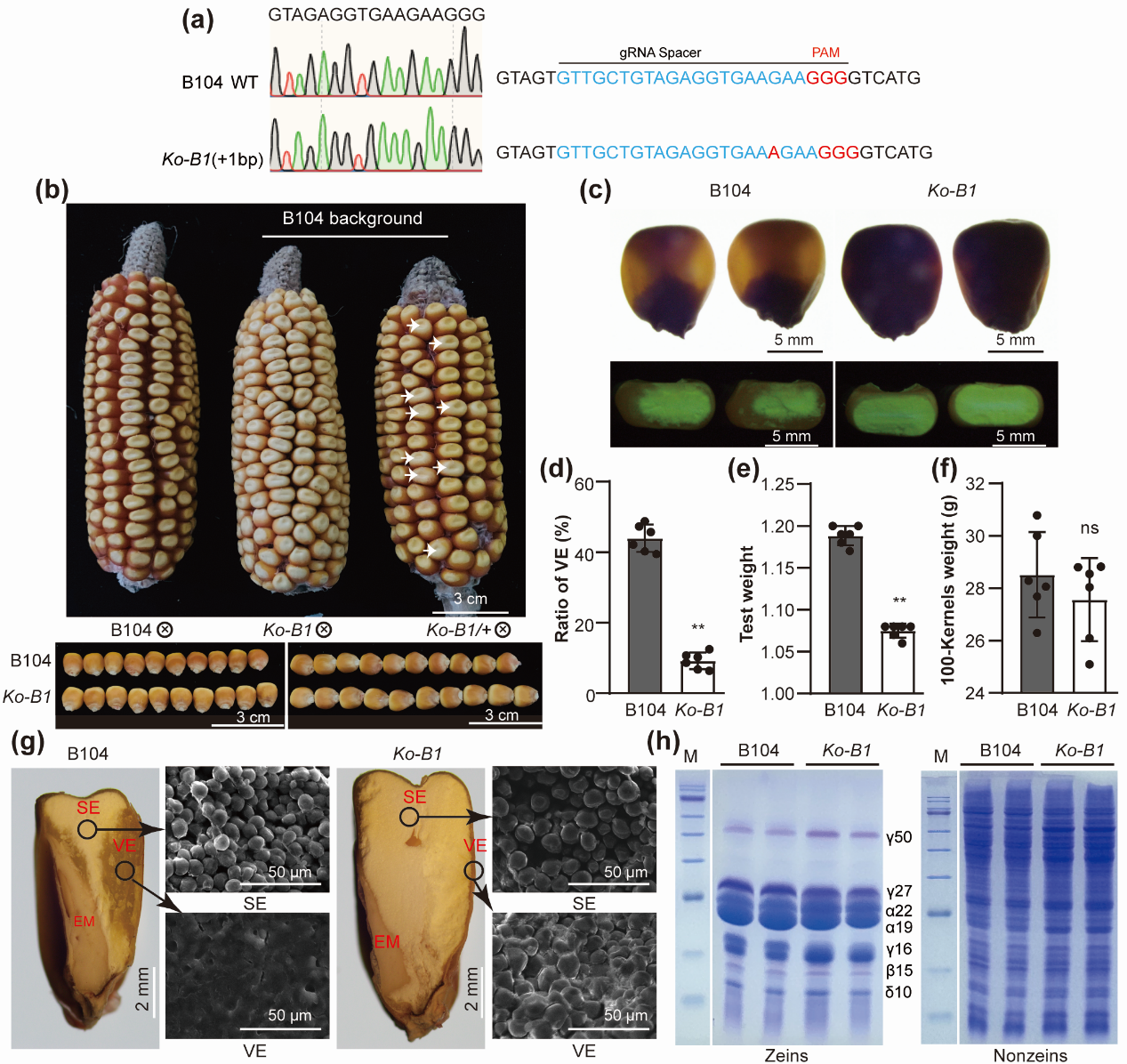


**Supplemental Figure 6. Kernel phenotypes of maize *zmmyb127* knock-out line in the B104 background.**

(a) Schematic diagram of the CRISPR/Cas9-edited sequence of *ZmMYB127*. The knockout line *Ko-B1* was recovered with 1 bp insertion in the B104 inbred line*.* (b) Ear and seed phenotypes of the WT B104 and *zmmyb127*. Upper panel, self-pollinated WT B104, *Ko-B1* and *Ko-B1*/+. The red arrowheads indicate the *zmmyb127* kernels. Lower panel, comparisons of the kernel length and width between WT B104 and *zmmyb127* mature seeds. (c) WT B104 and *zmmyb127* mature seeds observed in a light box (upper panel), and cross sections of WT B104 and *zmmyb127* mature seeds (lower panel). (d-f) Measurements of the ratio of VE (d), test weight (e) and 100-kernel weight (f). Data are presented as means ± s.d. Statistical significance (ns, not significant; **P* < 0.05; ***P* < 0.01) was determined by two-tailed Student’s *t*-test. (g) Scanning electron micrographs of the longitudinal section of the mature WT B104 and *zmmyb127* kernels. The black circle shows the location of the enlarged image in the right panel. (h) SDS-PAGE analysis of zein (left) and nonzein (right) proteins in WT B104 and *zmmyb127* mature kernels. M, marker. Total protein loaded in each lane was equal to 400 µg of mature kernel flour. The marker and sample lanes originated from the same gel but were rearranged for clarity.


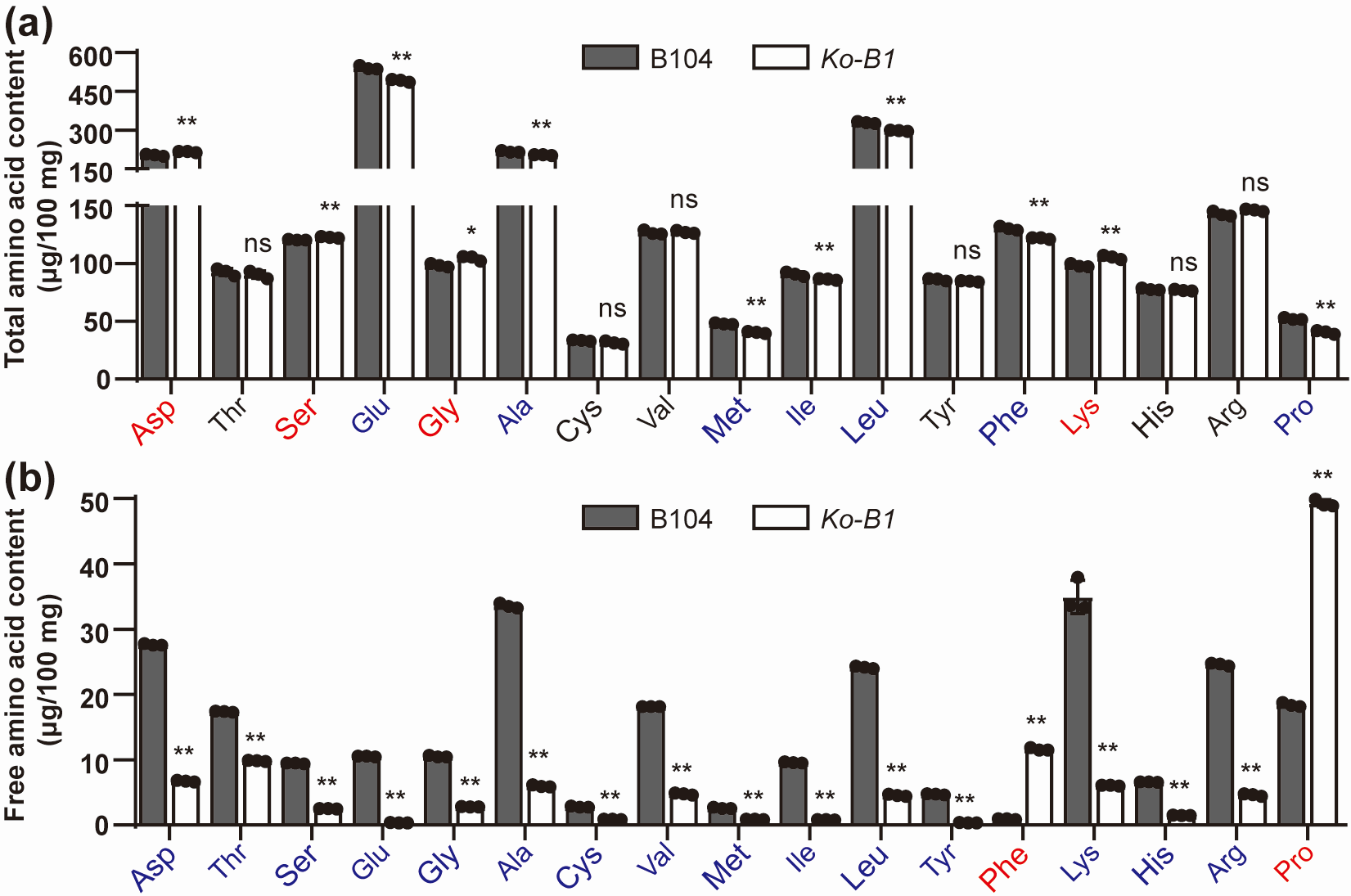


**Supplemental Figure 7. Measurement and comparison of total amino acid (TAA) and free amino acid (FAA) in WT and *zmmyb127* mature kernels.**

(a, b) TAA (a) and (b) and FAA contents of the WT and *zmmyb127* mature kernels. Data are presented as means ± s.d. (n=3). Statistical significance (ns, not significant; **P* < 0.05; ***P* < 0.01) was determined by two-tailed Student’s *t*-test.


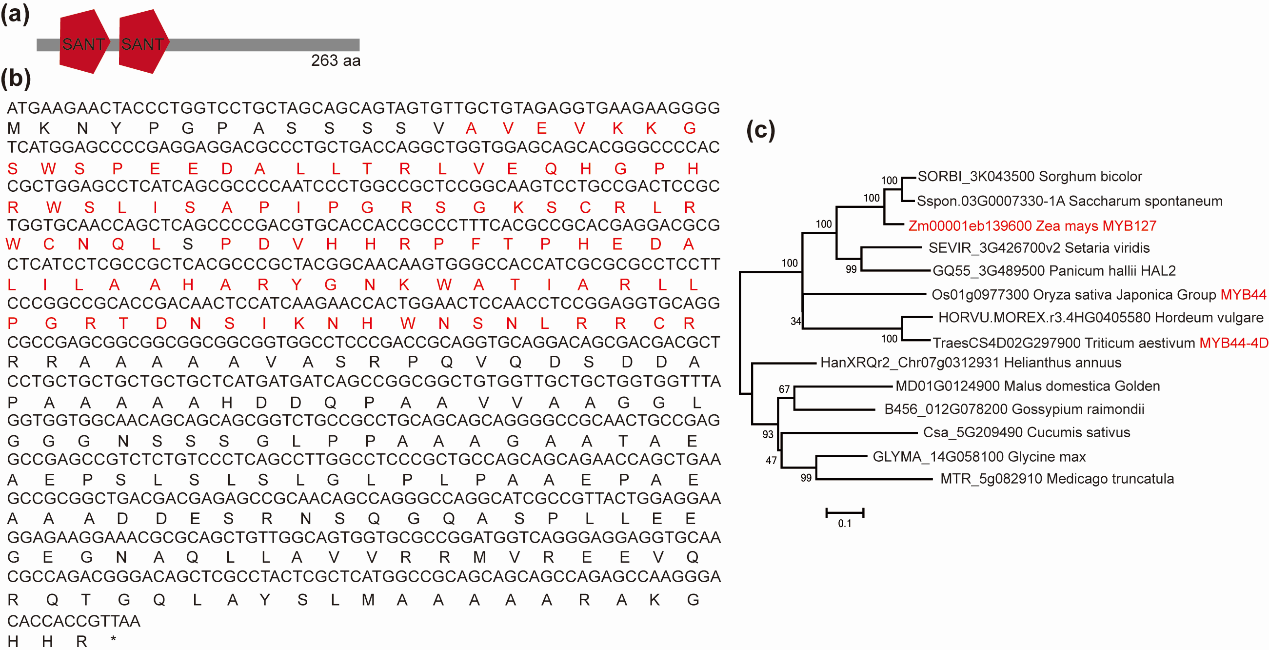


**Supplemental Figure 8. The primary sequence and phylogenetic analysis of ZmMYB127.**

(a) The conserved domain of ZmMYB127 protein was predicted in SMART (http://smart.embl-heidelberg.de/)*.* (b) The *ZmMYB127* gene sequence was cloned from maize bred line B73 and the translated amino acid sequence. The sequence marked in red represents conserved SANT domain. (c) The phylogenetic tree of ZmMYB127 and its homologs. The homologous proteins of ZmbZIP75 were obtained by NCBI (<https://www.ncbi.nlm.nih.gov/)> blast. Evolutionary distances were estimated with neighbor joining (NJ) method in MEGA (version 5.10). The bootstrap method with 1,000 replicates was applied for statistical testing. The ZmMYB127 was marked by red triangle label.


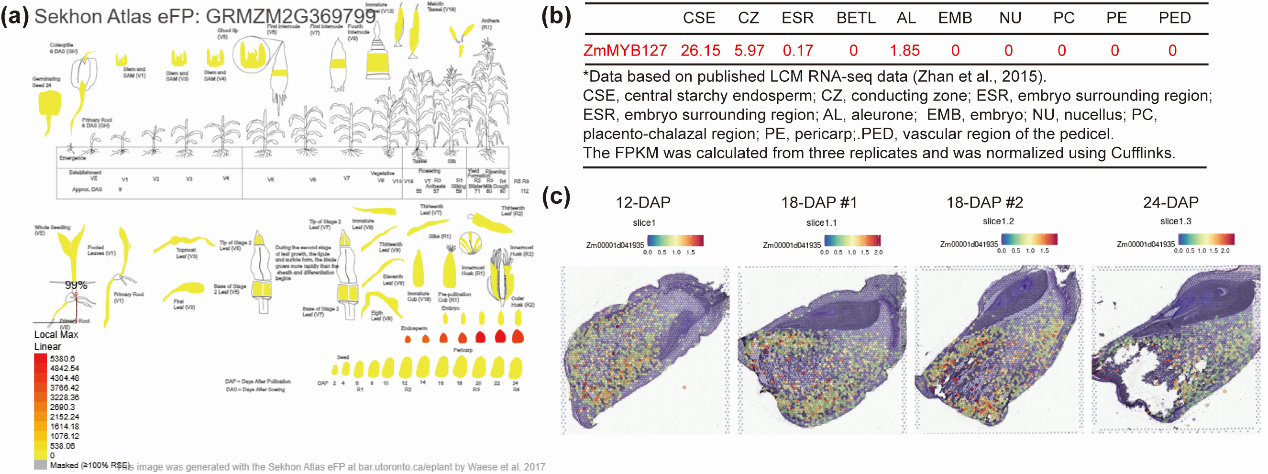


**Supplemental Figure 9. Expression pattern of *ZmMYB127*.**

(a) Temporal and spatial expression patterns of *ZmMYB127*. The relative intensity of color (grey, absent; yellow, low; red: high) indicating mRNA abundance in the indicated tissues. This image was generated from Maize eFP Browser ( <https://bar.utoronto.ca/eplant_maize/>) based on data from Hoopes *et al* (2019). (b) Normalized expression levels of *ZmMYB127* in different compartments of the maize kernel based on published LCM RNA-Seq data (Zhan *et al*., 2015). (c) Spatial expression patterns of *ZmMYB127* in maize kernels. The source data and heatmap were downloaded from Fu *et al* (2023).


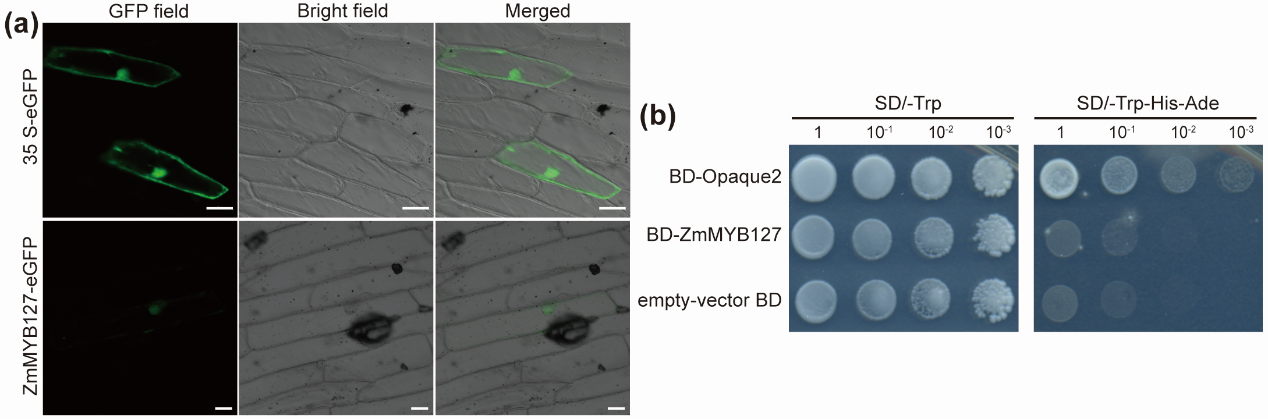


**Supplemental Figure 10. The** **functional characters of transcription factor ZmMYB127.**

(a) Subcellular localization of ZmMYB127. The construct containing the ZmMYB127:GFP fusion protein was transformed into onion epidermal cells via particle bombardment. 35S-eGFP was used as the control. GFP, green fluorescent protein. Bar = 50 μm. (b) Transactivation analysis of ZmMYB127 in yeast. BD (the pGBKT7 empty vector) was used as a negative control, and BD-Opaque2 was used as a positive control. SD, synthetic dropout medium.


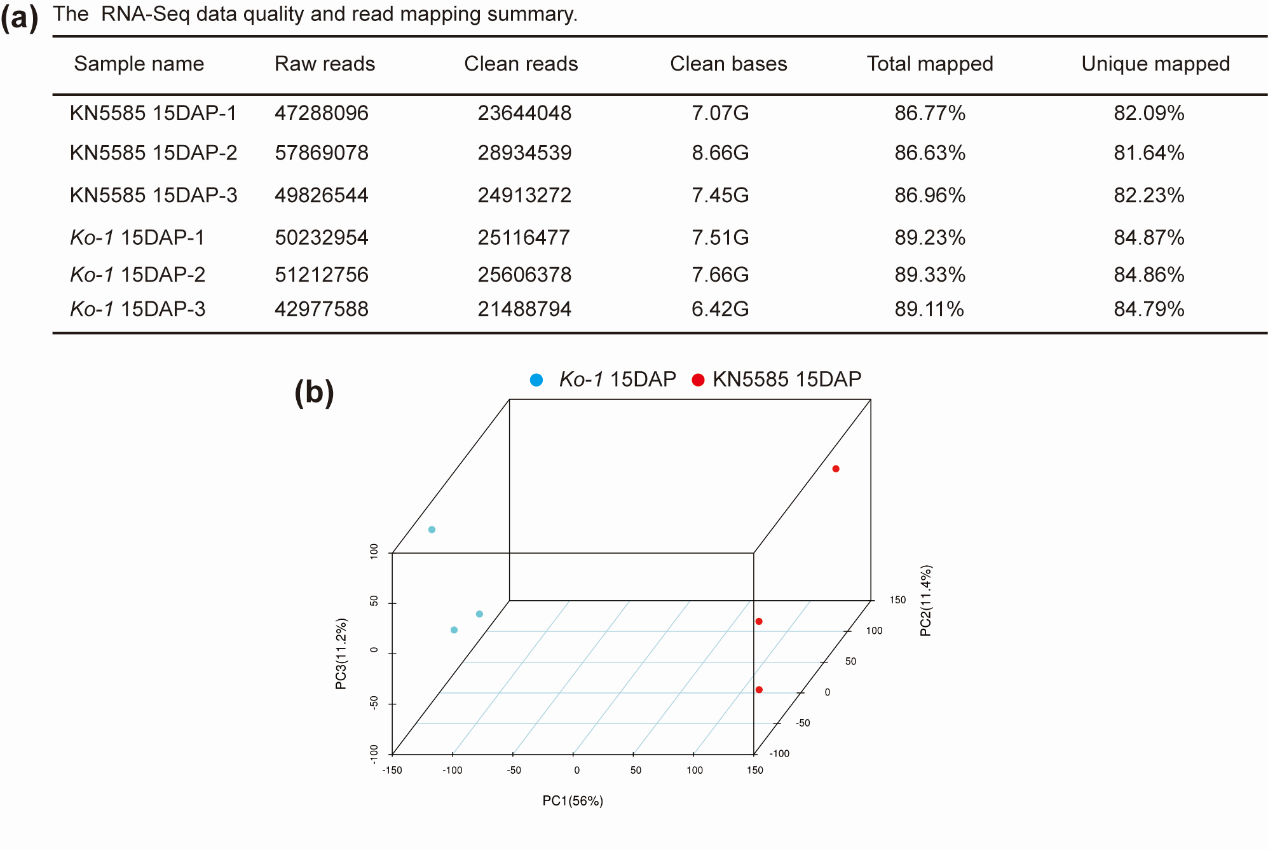


**Supplemental Figure 11. Characteristics of the RNA-Seq analysis.**

(a) Summary table of the RNA-Seq data quality and read mapping. (b) Principal component analysis of transcriptome data.


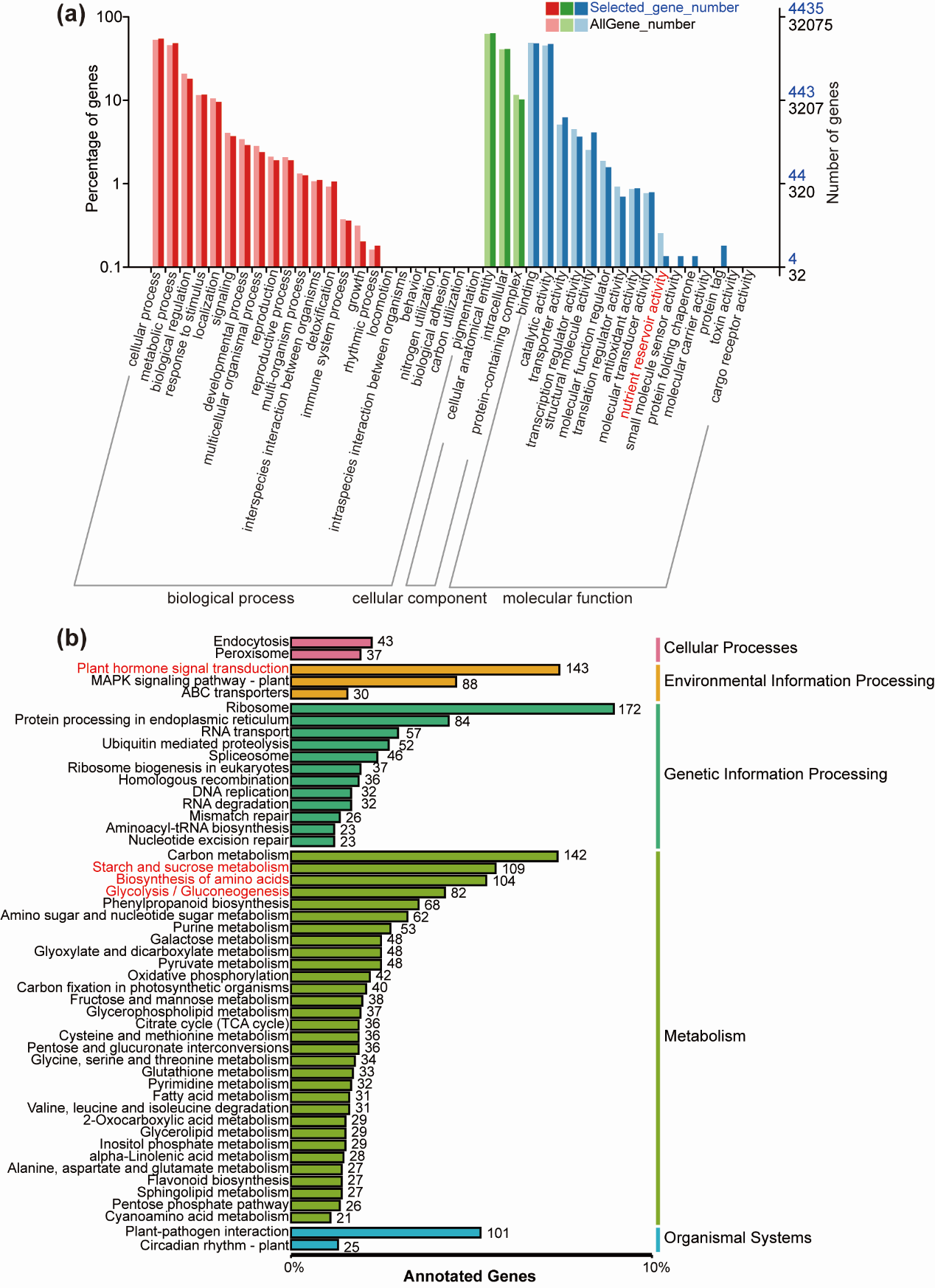


**Supplemental Figure 12. GO (a) and KEGG (b) enriched analysis of DEGs in *Ko-1* developing kernels versus WT KN5585 at 15-DAP.**


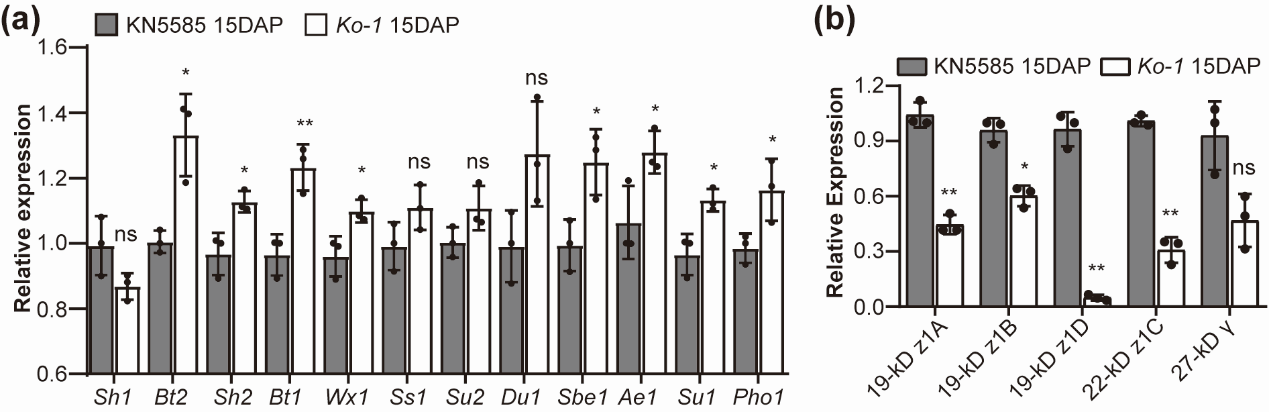


**Supplemental Figure 13. RT-qPCR analysis of starch synthesis genes (a) and zein gene expression (b) in the 15-DAP kernels of WT KN5585 and *Ko-1*.**

All relative expression levels were normalized to those of *Actin*. Data are presented as the means ± s.d. (n=3). Statistical significance (ns, not significant; **P* < 0.05; ***P* < 0.01) was determined by two-tailed Student’s *t*-test.


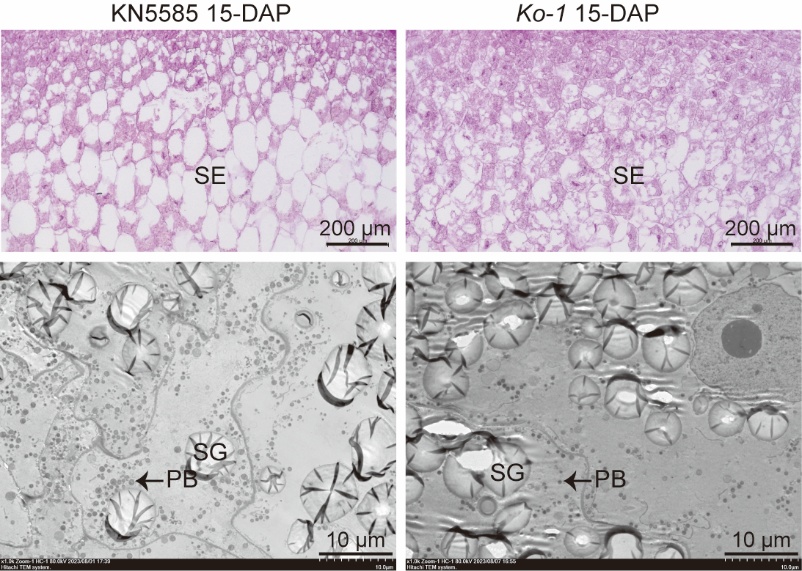


**Supplemental Figure 14. Light microscopy observations (upper panel) and transmission electron microscopy images (lower panel) of 15-DAP endosperm of the WT KN5585 and *zmmyb127* at the starchy endosperm (SE).**

PB protein body; SG starch granule.

**
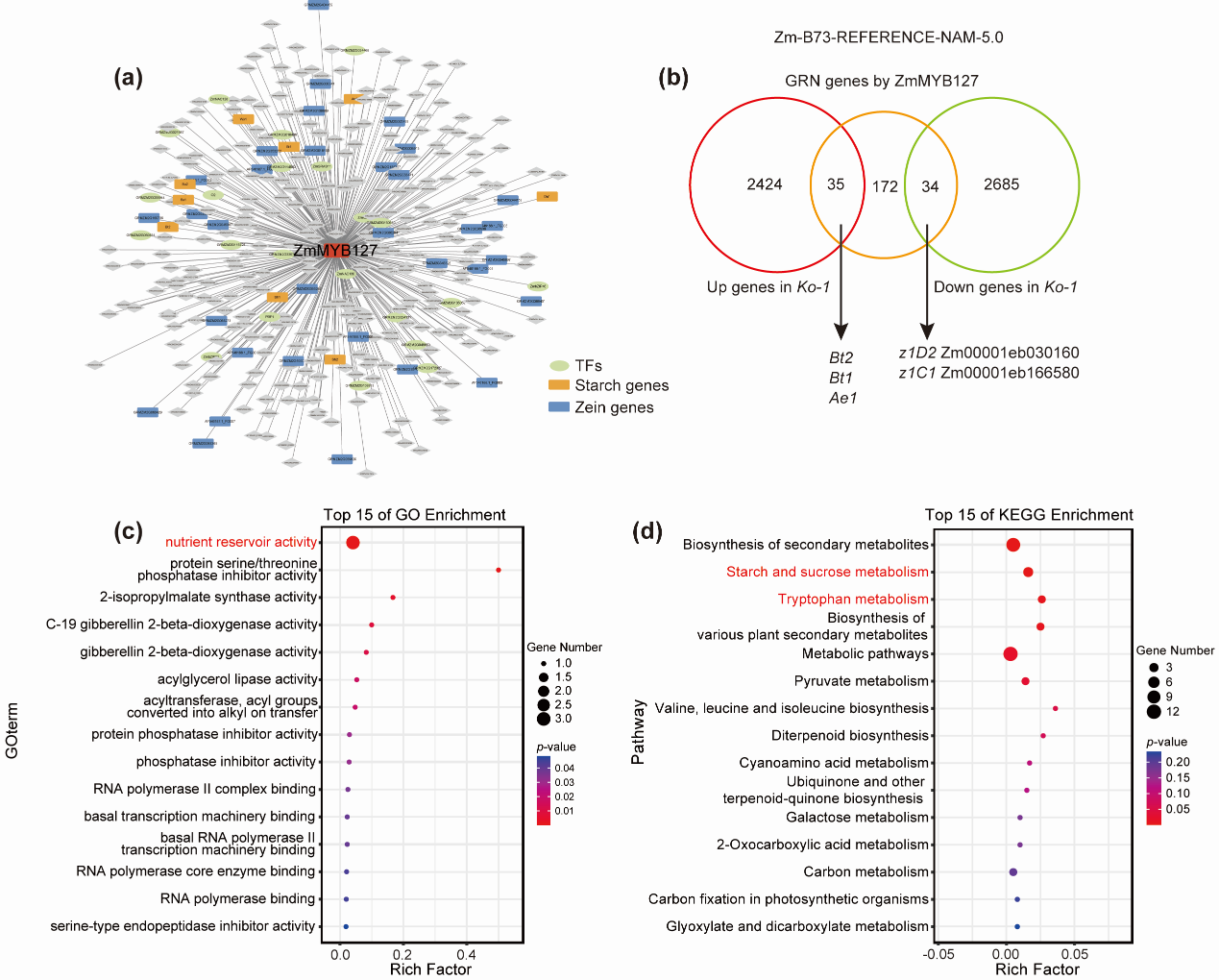
**

**Supplemental Figure 15. Analysis of potential target genes of ZmMYB127.**

(a) The gene regulatory network (GRN) of ZmMYB127. Transcription factors (TFs), starch synthesis and zeins expressed genes are colored in green, orange and blue, respectively. (b) Venn diagram showing the relationship between these genes of GRN by ZmMYB127 and all DEGs in *Ko-1* developing kernels versus WT at 15-DAP. (c, d) GO (c) and KEGG (d) enriched analysis of target genes of ZmMYB127.


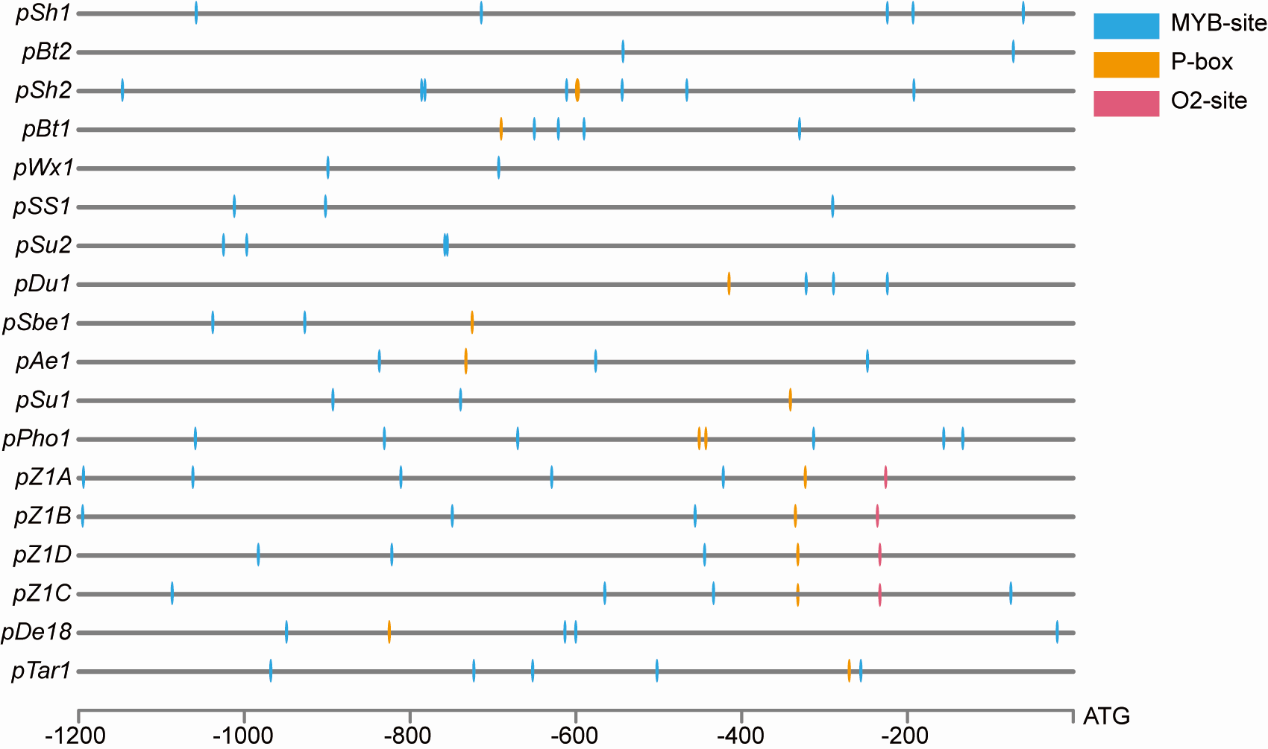


**Supplemental Figure 16. Analysis of MYB-binding sites, PBF1-binding sites and O2-binding sites in the promoters of core SSGs, major zein genes and IAA synthesis genes.**

The 1200 bp upstream of the start codons of starch synthesis genes were used for sequence analysis, and the MYB-binding sites were predicted by PlantCARE. The PBF1-binding sites and O2-binding sites were annotated based on previous studies (Ning *et al.*, 2023; Yang *et al.*, 2016).


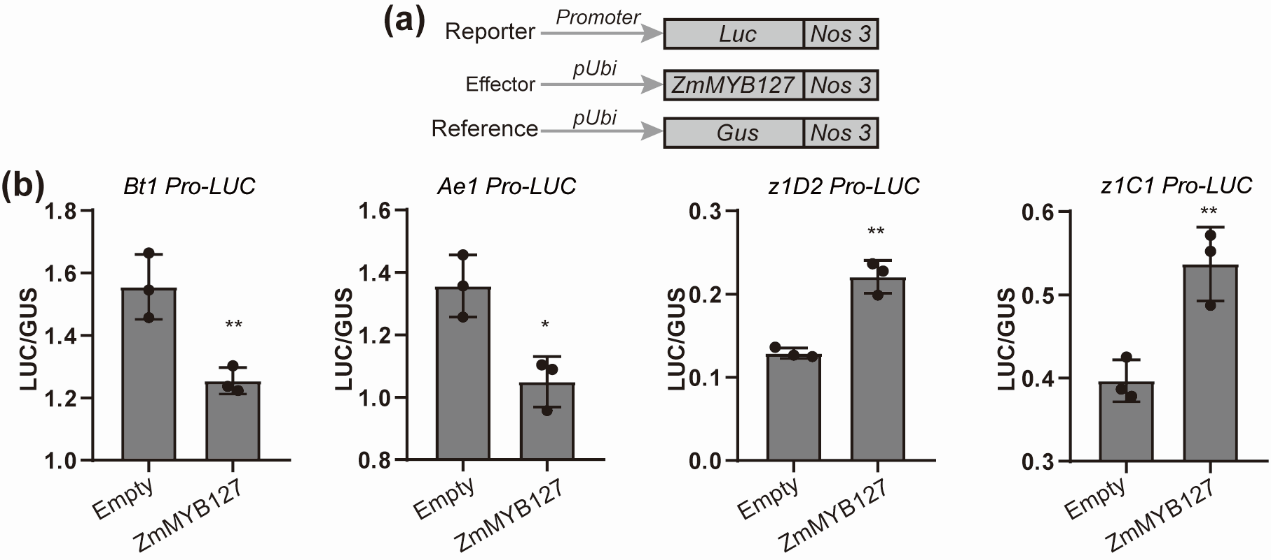


**Supplemental Figure 17. Effect of ZmMYB127 transient expression on the promoter activity of *Bt1*, *Ae1, z1D2 and z1C1* genes via maize leaf protoplasts.**

The LUC/GUS ratio represents the relative activity of target promoters. Data are presented as means ± s.d. (n=3). Statistical significance (**P* < 0.05; ***P* < 0.01) was determined by two-tailed Student’s *t*-test.


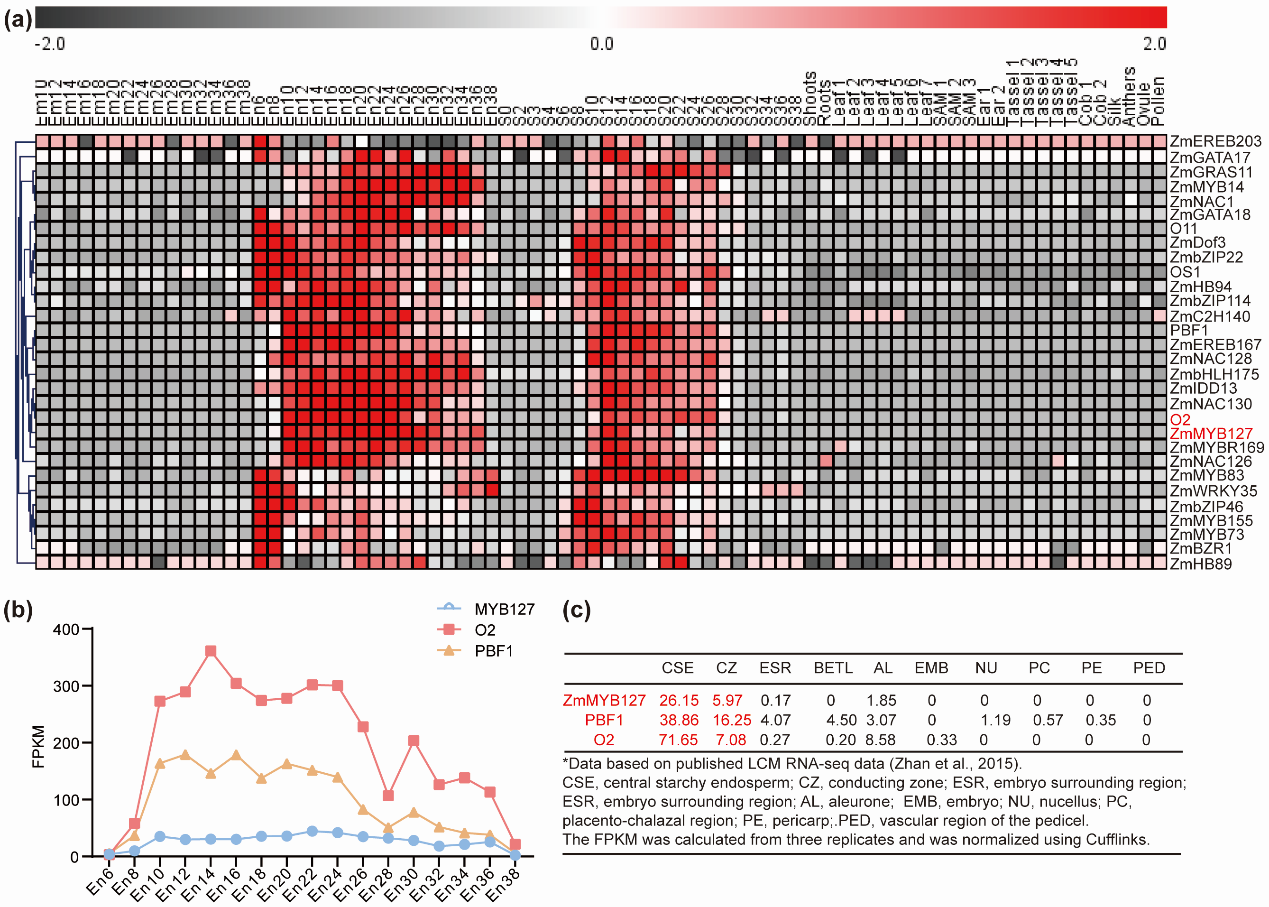


**Supplemental Figure 18. Expression pattern relationships between ZmMYB127, PBF1 and O2.**

(a) Heatmap showing the expression pattern of these specifically or highly expressed TFs in endosperm based on the public RNA-Seq data (Chen *et al.*, 2014). (b) The expression levels of *ZmMYB127*, *PBF1* and *O2* during endosperm development. The expression patterns were downloaded from the published database (Chen *et al.*, 2014). (c) Normalized expression levels of *ZmMYB127*, *PBF1* and *O2* in different compartments of the maize kernel based on published LCM RNA-Seq data (Zhan *et al.*, 2015).

**
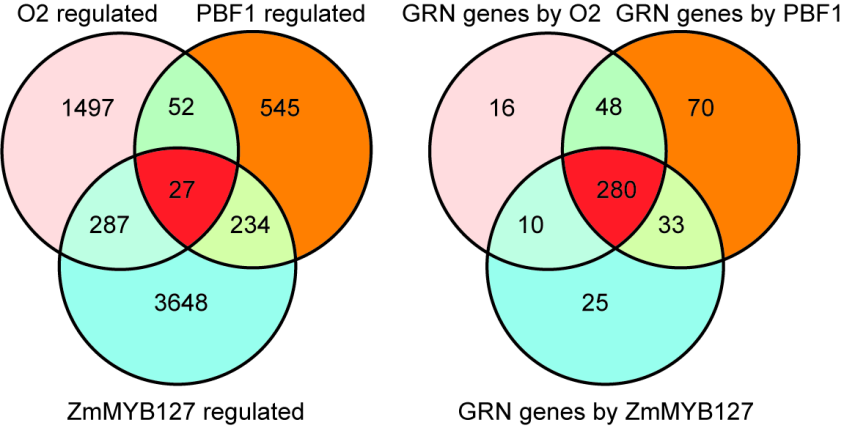
**

**Supplemental Figure 19. Venn diagram showing the relationships between ZmMYB127 regulated genes, PBF1 regulated genes and O2 regulated genes.**

The O2 regulated genes were from published RNA-Seq data (Zhan *et al.*, 2018), and PBF1 regulated genes were from published RNA-Seq data (Ning *et al.*, 2023).

**
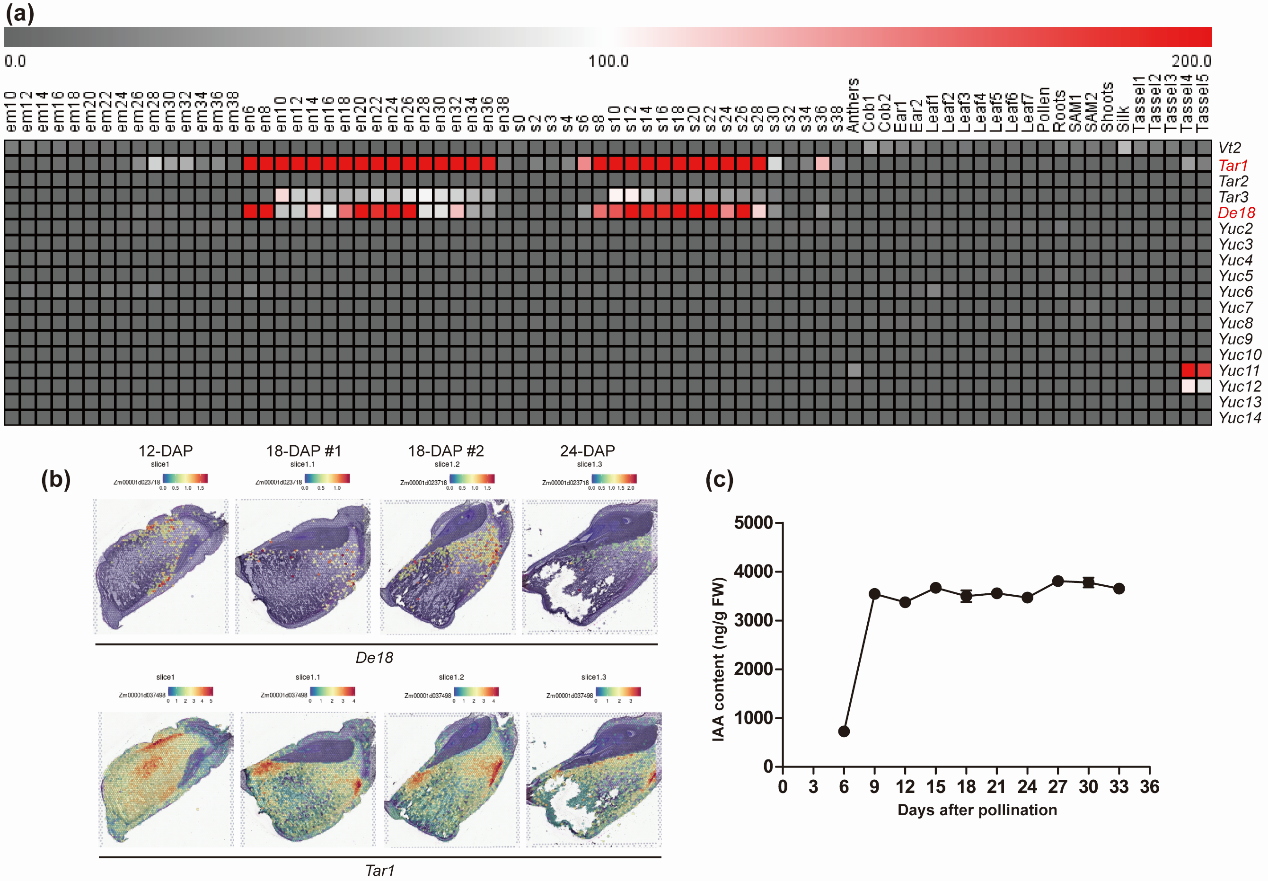
**

**Supplemental Figure 20. IAA synthesis in maize kernel.**

(a) Heatmap showing the expression patterns of IAA synthesis genes based on the public RNA-Seq Data (Yi *et al.*, 2019). (b) Spatial expression patterns of *De18* and *Tar1* in maize kernel. The source data and heatmap were downloaded from Fu *et al* (2023). (c) Concentrations of indole-3- acetic acid (IAA) in developing maize kernels of B73. Data are presented as means ± s.d. (n=3).

**References**

Yi F, Gu W, Chen J, Song N, Gao X, Zhang X, Zhou Y, Ma X, Song W, Zhao H, Esteban E, Pasha A, Provart NJ and Lai J (2019) High Temporal-Resolution Transcriptome Landscape of Early Maize Seed Development. *The Plant Cell* **31**:974-992.
